# CD34 defines melanocyte stem cell subpopulations with distinct regenerative properties

**DOI:** 10.1371/journal.pgen.1008034

**Published:** 2019-04-24

**Authors:** Sandeep S. Joshi, Bishal Tandukar, Li Pan, Jennifer M. Huang, Ferenc Livak, Barbara J. Smith, Theresa Hodges, Anup A. Mahurkar, Thomas J. Hornyak

**Affiliations:** 1 Department of Biochemistry and Molecular Biology, University of Maryland School of Medicine, Baltimore, Maryland, United States of America; 2 Department of Microbiology and Immunology, University of Maryland School of Medicine, Baltimore, Maryland, United States of America; 3 Marlene and Stuart Greenebaum Comprehensive Cancer Center, University of Maryland School of Medicine, Baltimore, Maryland, United States of America; 4 Institute for Basic Biomedical Sciences, John Hopkins School of Medicine, Baltimore, Maryland, United States of America; 5 Institute for Genome Sciences, University of Maryland School of Medicine, Baltimore, Maryland, United States of America; 6 Research & Development Service, VA Maryland Health Care System, United States Department of Veterans Affairs, Baltimore, Maryland, United States of America; 7 Department of Dermatology, University of Maryland School of Medicine, Baltimore, Maryland, United States of America; Stanford University School of Medicine, UNITED STATES

## Abstract

Melanocyte stem cells (McSCs) are the undifferentiated melanocytic cells of the mammalian hair follicle (HF) responsible for recurrent generation of a large number of differentiated melanocytes during each HF cycle. HF McSCs reside in both the CD34+ bulge/lower permanent portion (LPP) and the CD34- secondary hair germ (SHG) regions of the HF during telogen. Using *Dct-*H2BGFP mice, we separate bulge/LPP and SHG McSCs using FACS with GFP and anti-CD34 to show that these two subsets of McSCs are functionally distinct. Genome-wide expression profiling results support the distinct nature of these populations, with CD34- McSCs exhibiting higher expression of melanocyte differentiation genes and with CD34+ McSCs demonstrating a profile more consistent with a neural crest stem cell. In culture and *in vivo*, CD34- McSCs regenerate pigmentation more efficiently whereas CD34+ McSCs selectively exhibit the ability to myelinate neurons. CD34+ McSCs, and their counterparts in human skin, may be useful for myelinating neurons *in vivo*, leading to new therapeutic opportunities for demyelinating diseases and traumatic nerve injury.

## Author summary

The hair follicle (HF) undergoes three different stages, anagen, catagen, and telogen during each hair cycle. In anagen, melanocyte stem cells (McSCs) give rise to differentiated melanocytes which are responsible for coloration of hair. In catagen, melanocytes undergo apoptosis while McSCs are retained. In the resting telogen HF, McSCs are identified as non-proliferating and quiescent populations. Interestingly, in a mouse model, we identified McSCs in both CD34+ bulge and CD34- secondary hair germ (SHG) compartments of telogen HFs. In this study, we separated and characterized McSC subpopulations from these two distinct compartments of telogen HFs. Using *Dct*-H2BGFP mice, bulge and SHG McSCs were separated using CD34. Based on genomic approaches and functional assays we found that CD34- McSCs (SHG) are primed for melanocyte differentiation and CD34+ McSCs (bulge) exhibit broader neural crest stem cell properties and demonstrate ability to differentiate into glia and myelinate neurons. Our results thus reveal functional heterogeneity of McSC subtypes.

## Introduction

Neural crest-derived melanocyte stem cells (McSCs) are responsible for producing differentiated melanocytes during each hair follicle (HF) cycle. During embryogenesis, neural crest cells emerging from neural tube generate melanoblasts which migrate to specific destinations including eye, epidermis and developing HF where they continue to proliferate and produce pigment-producing melanocytes in early postnatal life. In growing HFs, McSCs are distinguished from hair matrix melanocytes by their location in the outer root sheath (ORS) of the bulge/ lower permanent portion (LPP) and by distinct molecular signatures, including the expression of Dct and Pax3, but low Sox10 [1]. In resting HFs, McSCs are identified based on their quiescence properties, expression of Dct, and by their localization within HF bulge/LPP and a region previously described as the subbulge region [2].

The HF is a skin appendage composed of epithelial cells, follicular cells, mesenchymal cells and pigment-producing melanocytes. During each cyclic expansion and regression, the mammalian HF proceeds through three distinct phases, anagen (growth phase), catagen, (regression phase), and telogen, (a follicular resting phase) [3, 4]. The cycle is initiated for follicular expansion when HF stem cells (HFSCs) in the hair germ of telogen HFs are activated by factors including noggin (NOG), FGF-7, FGF-10 and TGF-β2 secreted from dermal papillae [5, 6]. In the newly-initiated cycle, McSCs become activated by a Wnt signal from HFSCs in the surrounding vicinity to generate proliferating, committed melanocyte progenitors [7]. During fully-developed anagen, terminally-differentiated melanocytes reside in the inner core of the hair matrix, where they produce and transfer melanin to the surrounding follicular epithelial cells. During catagen, melanocytes degenerate along with the rest of the matrix and lower ORS [8] and the HF returns to the resting phase. The quiescent state of telogen HFSCs is maintained by factors including BMP6 and FGF-18 from inner bulge cells [9], BMP4 from dermal fibroblasts and BMP2 from subcutaneous adipocytes [10]. In telogen, the lower HF component consists of two regions, the bulge/LPP [11] and secondary hair germ (SHG) [12], an epithelial extension at the base of the telogen HF. These compartments can be distinguished using region-specific markers, with the bulge compartment expressing the CD34 membrane glycoprotein [13] and the SHG selectively expressing the intracellular adhesion protein P-cadherin/Cdh3 [14]. McSCs along with other HFSCs are present in both compartments.

Recently, by using tetracycline-regulated expression of a stable H2BGFP fusion protein from the *Dct* promoter in bitransgenic *Dct-*tTA;TRE-H2BGFP (*Dct-*H2BGFP) mice, we localized subbulge McSCs to the SHG, a transient structure at the base of the telogen HF [15]. Given this localization of McSCs to anatomically separate telogen HF compartments whose epithelial stem cells possess distinct characteristics, we wondered whether McSCs occupying these distinct sections were functionally different or interchangeable. We describe for the first time that McSCs can be separated into two distinct populations, corresponding to cells present in the bulge and SHG HF compartments, using CD34. We show that CD34+ McSCs from the HF bulge unexpectedly possess the ability to function as glia, forming dense myelin sheaths surrounding neurons of the myelin-deficient *Shiverer* mouse strain. They function less efficiently as McSCs compared to CD34- McSCs. This finding raises the question of whether all cells previously identified as McSCs uniformly possess melanocytic potential, or whether the CD34+ subset of these cells represents instead another type of neural crest-derived progenitor cell. Our findings reveal a novel developmental fate for one subset of McSCs, and suggest approaches to utilize specific, skin-derived stem cell (SDSC) populations for nerve regeneration and support.

## Results

### Identification of GFP-expressing McSCs in bulge and SHG of telogen HF of *Dct*-H2BGFP bitransgenic mice

**Fig 1:**
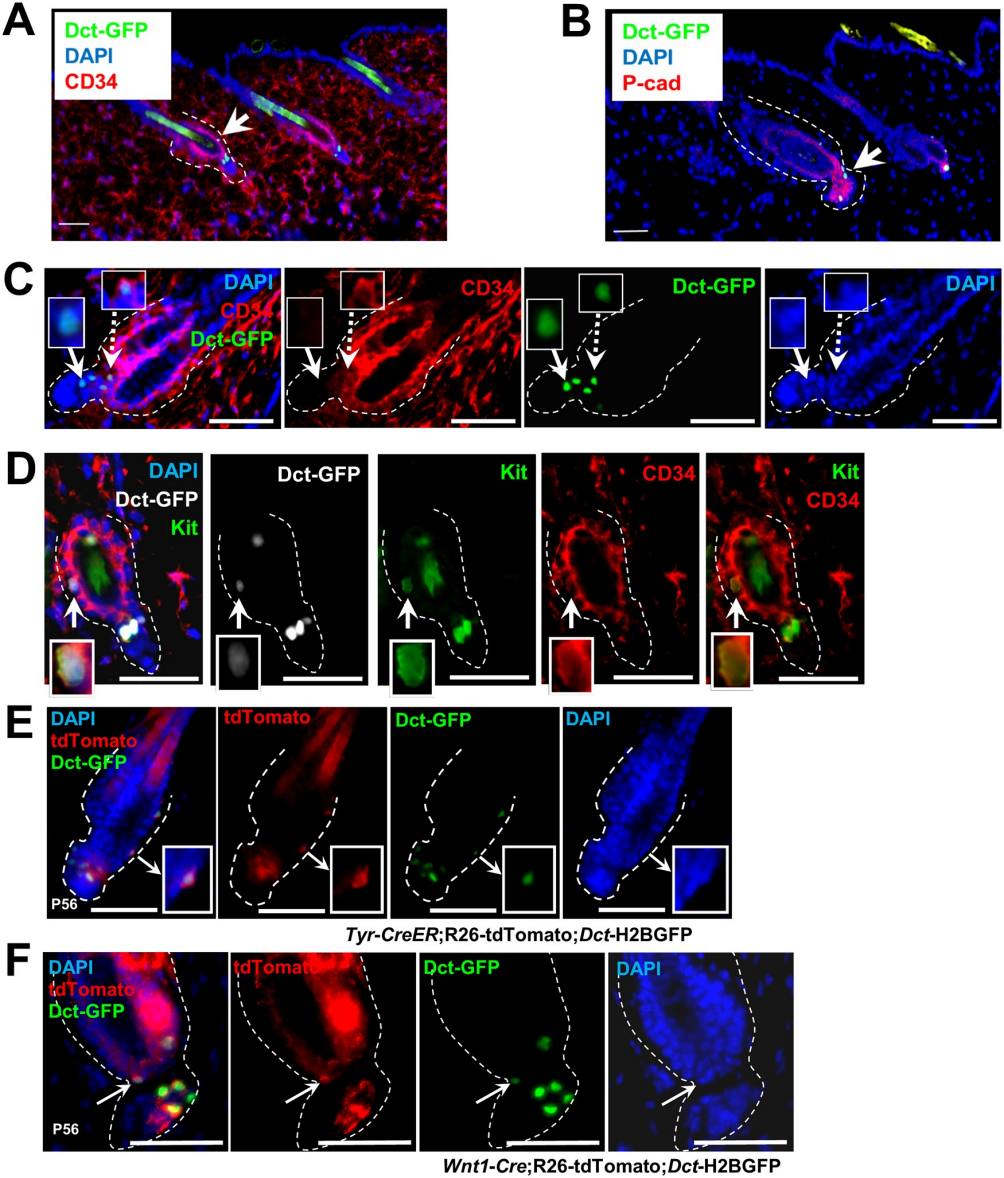
Identification of GFP-expressing melanocyte precursor cells in bulge and SHG of telogen HF. Distinct subpopulations of GFP-expressing cells in the CD34+ bulge region (arrows, A) and P-Cad+ SHG region (arrows, B) in P56 dorsal skin HFs. Scale bars: (A) 100 μm (B) 50 μm. (C) GFP-expressing cells and anti-CD34 immunofluorescence in P56 dorsal skin HF. Dotted arrow depicts co-localization of bulge GFP-expressing cell and CD34 expression and solid arrow shows SHG GFP-expressing cells lack CD34 expression. Scale bars: 50 μm. (D) Immunofluorescence of Kit and CD34 expression in bulge GFP-expressing cells in P56 dorsal skin HF. Arrow depicts co-localization of bulge GFP-expressing cell with Kit and CD34 expression. Scale bars: 25 μm. (E & F) Co-localization of *Tyr* (E) or *Wnt1* (F) driven tdTomato expression and GFP-expressing cells from both bulge and SHG (arrows) in telogen HF of *Tyr-CreER*;R26-tdTomato;*Dct*-H2BGFP or *Wnt1*-Cre;R26-tdTomato;*Dct*-H2BGFP mice at P56 respectively. Scale bars: 50 μm.

To identify McSCs in telogen HFs, we used *Dct*-H2BGFP bitransgenic mice [15]. The constitutive expression of *Dct*-H2BGFP mice is comparable to the expression pattern of *Dct*-LacZ [2, 16, 17] and *iDct*-GFP mice [18], which express the transgene in melanoblasts, melanocyte progenitors in bulge/LPP and terminally differentiated melanocytes [15]. *Dct*-H2BGFP cells were present in both the CD34-expressing bulge region [9] of the HF and the CD34-negative, P-cadherin-expressing SHG region [6] at the base of the telogen HF (Fig 1A and 1B, S1A–S1D Fig). *Dct*-H2BGFP cells in second telogen express the McSC markers Kit and Dct [15]. Careful observation of *Dct-*H2BGFP cells in the bulge HF region revealed not only that these cells were present in the CD34+ region, but also express CD34 (Fig 1C). To confirm that CD34 expression was not limited to the epithelial cells of the bulge, we showed that Kit, which is restricted to HF melanocytes, and CD34 are co-localized (Fig 1D). Given the distinction between *Dct-*H2BGFP cells either expressing CD34 in the bulge or lacking CD34 in the SHG, we determined formally whether each of these cell classes was neural crest-derived and from the melanocytic lineage. *Dct-*H2BGFP cells co-localize both with a *Tyr-CreER*;R26-tdTomato signal (Fig 1E) and with a *Wnt1*-Cre;R26-tdTomato signal (Fig 1F), demonstrating that CD34+ and CD34- *Dct-*H2BGFP cells originate from neural crest and are members of the melanocyte lineage. Since our identification of CD34+ and CD34- cells as putative distinct populations of McSCs was limited to the telogen phase, we also attempted to identify CD34+ McSCs selectively throughout the HF cycle. These cells could be identified at the follicular morphogenesis stage (P8), at the first telogen (P21), and during anagen stages at both P30 and P70 (S2A–S2F Fig). CD34- McSCs were also present at the P21 telogen stage. The SHG does not remain as a recognizable anatomic HF feature during anagen, making it difficult to establish whether individual cells beneath the bulge during these stages retain this identity during anagen. Nonetheless, analysis of expression of the differentiated melanocyte markers Tyr and Tyrp1 in HF anagen at P30 shows a clear difference of expression in differentiated bulb melanocytes compared to bulge McSCs where they are not detectable at this stage (S3A and S3B Fig). The persistence of CD34+ *Dct*-H2BGFP cells throughout the HF cycle fulfills an important criterion for these cells as McSCs.

**Fig 2:**
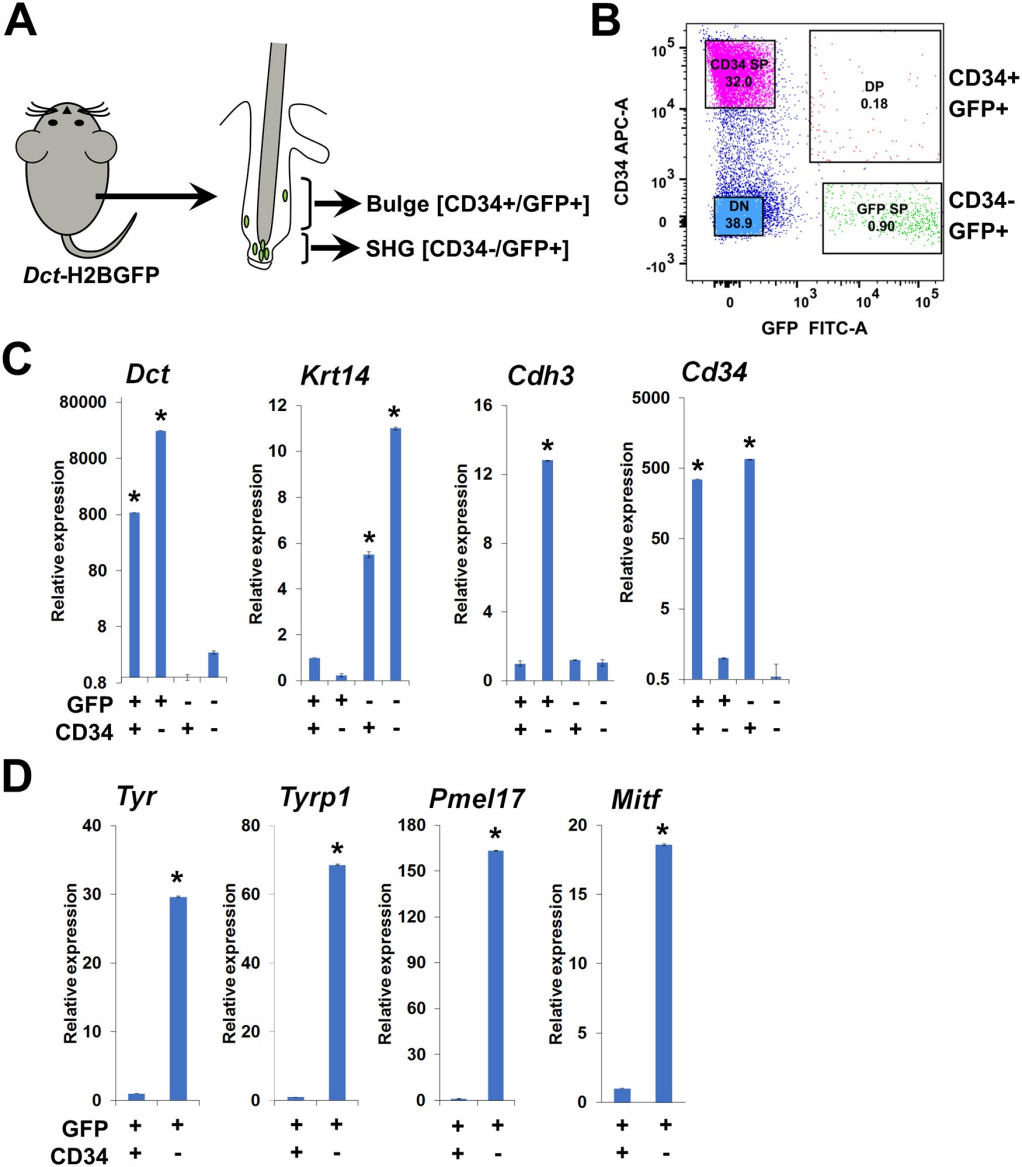
Separation of bulge and SHG GFP-expressing melanocyte precursor cells of telogen HFs. (A) Experimental scheme: McSCs identified in HF bulge and SHG of *Dct*-H2BGFP mice were separated using FACS with GFP and anti-CD34. (B) Separation of bulge (CD34+GFP+) and SHG (CD34-GFP+) melanocyte precursor cells and CD34+GFP- and CD34-GFP- dermal cells using FACS with GFP and anti-CD34, showing a representative image of the FACS. DP = Double positive, SP = Single positive and DN = Double negative. (C) Quantitative RT-PCR analysis for the expression of *Dct*, *Krt14*, *Cdh3*, and *Cd34* genes among the CD34+GFP+(bulge), CD34-GFP+(SHG), CD34+GFP- and CD34-GFP- sorted cell populations. Here, *Gapdh* was used as reference gene, (*P ≤ 0.01 by ANOVA). (D) Quantitative RT-PCR analysis for the expression of *Tyr*, *Tyrp1* and *Pmel17* in bulge (CD34+GFP+) and SHG (CD34+GFP+) sorted cells. Here, *Gapdh* was used as reference gene. (*P ≤ 0.01 by ANOVA).

Selective expression of CD34 by bulge *Dct-*H2BGFP cells suggested a strategy to separate these cells from SHG *Dct-*H2BGFP cells to evaluate their molecular and functional properties. Single cell suspensions prepared from shaven, dorsal skin of approximately 8-week-old (P56) mice, an age when all HFs are synchronously in the telogen [4], were incubated with anti-CD34 antibody and prepared (Fig 2A) for fluorescence-activated cell sorting (FACS). *Dct-*H2BGFP cells could be separated into distinct CD34+ and CD34- populations using FACS. Although the percentage yield of cells comprising these populations differed slightly between experiments, in general 0.1–0.3% of the dermal cell suspension was comprised of CD34+ and 0.5–1.0% CD34- *Dct-*H2BGFP cells (Fig 2B, S1E and S1F Fig). To further evaluate the specificity of these cell populations, RNA was isolated (S1G Fig), and relative gene expression for specific marker genes determined (Fig 2C). These results confirmed that *Dct-*H2BGFP cells expressed endogenous *Dct* at significantly higher levels than the basal keratinocyte gene *Krt14*, with *Dct* expression marginally higher in CD34- *Dct-*H2BGFP cells compared to CD34+ counterparts. Furthermore, *Cd34* expression was significantly higher in the CD34+ *Dct-*H2BGFP cells, with expression of *Cdh3*, encoding P-cadherin, reciprocally elevated in the CD34- *Dct-*H2BGFP cells corresponding to the P-cadherin-expression in the SHG population. To further evaluate the specificity of CD34+ and CD34- *Dct*-H2BGFP isolated cells, we examined FACS-separated cells individually for Kit and CD34 expression with immunofluorescence. CD34+ and CD34- *Dct-*H2BGFP McSCs expressed Kit, a melanocyte marker (S4A Fig), whereas CD34+ was only detected in CD34+GFP+ bulge McSCs and CD34+GFP- cells (S4B Fig). The epithelial HF stem cell marker Krt14 was only expressed in GFP- cells (S4C Fig).

### Bulge and SHG *Dct-*H2BGFP cells are McSCs with distinct functional properties

**Fig 3:**
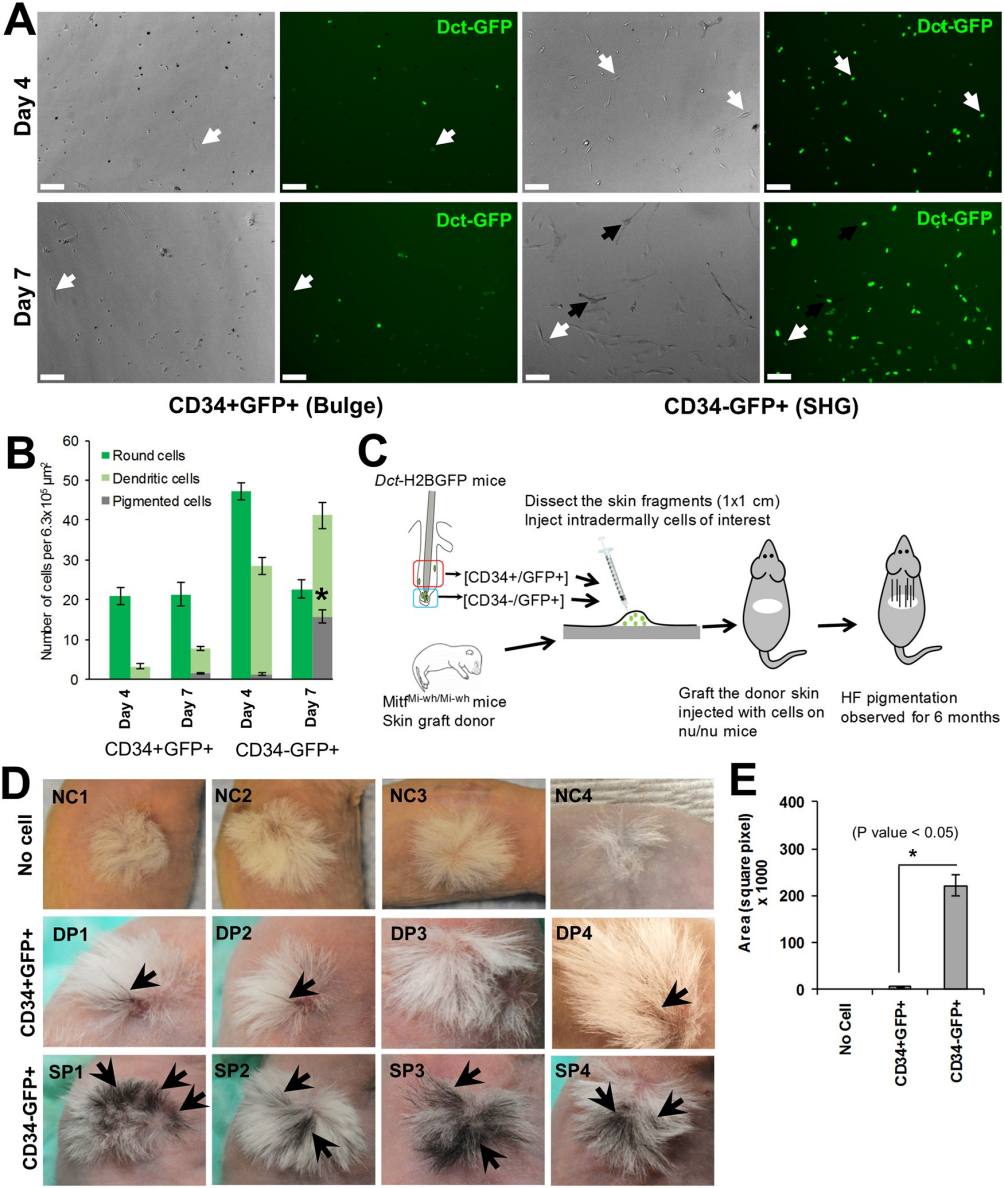
Distinct melanogenic properties of bulge and SHG melanocyte precursor cells of telogen HFs. (A) *In vitro* melanocyte differentiation potential of CD34+GFP+ and CD34-GFP+ McSCs in melanocyte differentiation culture condition for day 4 (top two panels) or day 7 (bottom two panels). Dark arrow indicates pigmented cells and white arrow indicates dendritic but non-pigmented cells. Scale bars: 100 μm. (B) Quantification of bulge and SHG McSCs potential to produce pigmented melanocytes in melanocyte differentiation medium at day 4 and 7. *P ≤ 0.01. (C) Viable CD34+GFP+ and CD34-GFP+ McSCs isolated from *Dct*-H2BGFP mice were injected into skin fragments of neonatal *Mitf*^*Mi-wh/Mi-wh*^ mice (P0 –P2). Injected skin fragments were engrafted onto nude mice to observe regeneration of HF pigmentation. (D) Skin grafts following reconstitution with CD34-GFP+ cells (SP) showed significantly greater regeneration of follicular pigmentation than grafts receiving CD34+GFP+ cells (DP). HF pigmented regions of the grafted skin are indicated by arrows. No cell (NC) injected grafted skin fragments were used as a negative control and showed no pigmented HFs. (E) Quantification of pigmented region of engrafted skin receiving CD34- McSCs, or CD34+ McSCs, or no cells. Area of pigmented region was calculated using Image J software. (* P Value < 0.05. by ANOVA; N = 5).

The ability to separate subsets of HF *Dct-*H2BGFP cells during telogen, together with their origin in the melanocytic lineage, prompted us to evaluate the relative expression of the melanogenic genes *Tyr*, *Tyrp1*, *Pmel17*, and *Mitf* within CD34+ and CD34- subsets. Quantitative RT-PCR results (Fig 2D) showed that relative expression of all four melanogenic genes studied was significantly higher in the CD34- McSCs present in the SHG compared to the CD34+ cells from the HF bulge, suggesting that the SHG McSCs are at a more advanced state of melanocytic differentiation than the cells in the bulge. To explore this finding functionally, cells were sorted, cultured in melanocyte differentiation medium, and observed after 4 and 7 days in culture. Only cultured cells in the CD34- *Dct-*H2BGFP cell culture exhibited visible pigmentation following these *in vitro* culture periods (Fig 3A). Quantification of cell pigmentation and morphology (Fig 3B) confirmed that significant numbers of pigmented cells only developed in the cultures of CD34- *Dct-*H2BGFP cells, with CD34+ *Dct-*H2BGFP cells principally maintaining a round, rather than dendritic, non-pigmented appearance even after 7 days of *in vitro* culture in melanocyte differentiation conditions. The percentage of pigmented cells from CD34+ cells increased from 0 to 5% from day 4 to day 7, while this percentage increased from 2% to 25% for CD34- cells introduced into the culture environment. Dendritic, non-pigmented cells also increased (CD34+, 13% to 27%; CD34-, 38% to 65%). Representative examples are shown in S5A Fig. CD34- McSCs also appeared to proliferate more in melanocyte differentiation medium than CD34+ McSCs (Fig 3B and S5B Fig). These findings provided evidence that CD34+ bulge *Dct-*H2BGFP cells are functionally distinct from the CD34- *Dct-*H2BGFP cell population.

We determined whether both CD34+ and CD34- *Dct-*H2BGFP cells, previously characterized as quiescent [15], were McSCs. We introduced subsets of cells into amelanocytic skin and observed transplanted skin for durable pigmentation. Equal numbers of CD34+ and CD34- McSCs isolated from *Dct*-H2BGFP telogen HFs were transplanted into *Mitf* ^*Mi-wh/Mi-wh*^ neonatal mouse skin which was then engrafted onto nude mice (Fig 3C). CD34- McSCs repigmented the amelanocytic murine HFs with high efficiency compared to CD34+ McSCs. 3/4 grafts of CD34+ cells and 4/4 grafts of CD34- cells demonstrated pigmentation at two months following graft placement. Pigmentation within the grafts was sustained, with durable pigmentation observed over 6 months. Pigmented foci were observed beginning at 2 weeks following engraftment, and in general did not expand or reduce in size over time. However, the pigmented foci were significantly larger following CD34- cell transfer compared to CD34+ cell transfer (Fig 3D and 3E). Following engraftment and successful *in vivo* reconstitution of either CD34+ or CD34- McSCs, cells are observed in both the CD34+ bulge and CD34- SHG, indicating at least a relative ability of each cell subset to populate both niches in this experimental context (S5C Fig). These results show that both CD34+ and CD34- *Dct-*H2BGFP are McSCs, but nonetheless they possess distinct differentiation profiles and markedly different efficiencies of McSC function.

### CD34+ bulge McSCs have characteristics of neural crest stem cells

**Fig 4:**
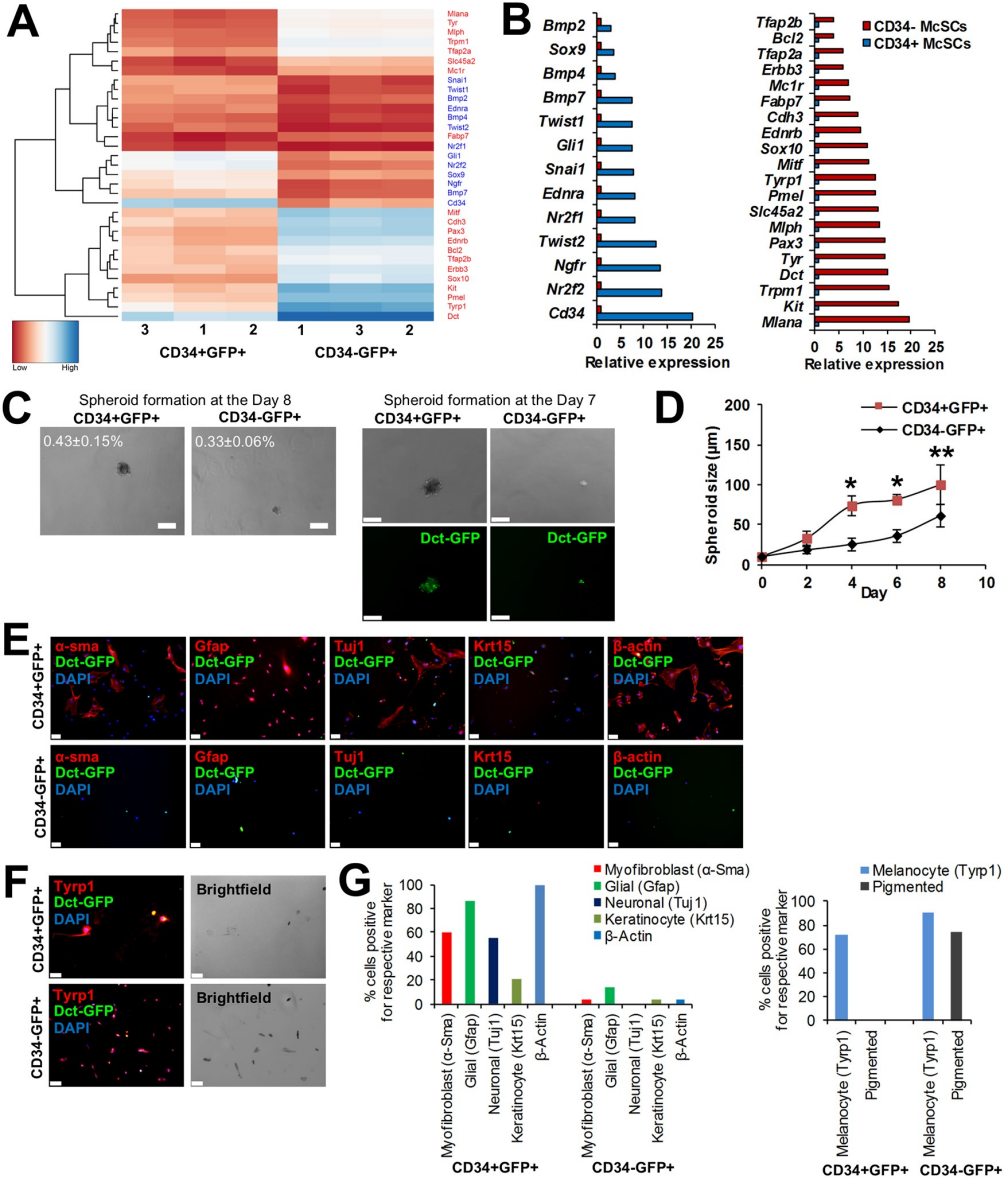
CD34+ bulge McSCs exhibit distinct neural crest lineage potential. (A) Heatmap of scaled and clustered, variance stabilizing transformation (VST) read count values obtained for select genes from RNA-seq analysis of CD34+ and CD34- McSCs. In bulge and SHG McSCs, the melanogenic marker (red) and NCSC marker (blue) are differentially expressed, closely clustered and are statistically significant. (P^adjusted^ < 0.02, Benjamini-Hochberg adjusted p value) (B) RNA-Seq analysis, based on fold change, showed that CD34+GFP+ McSCs (blue; left panel) express higher NCSC markers, whereas CD34-GFP+ McSCs (red; right panel) express higher melanogenic markers. (C) Formation of non-adherent spheroids was studied among CD34+GFP+ (bulge) and CD34-GFP+ (SHG) McSCs when cultured in NCSC medium (top panels). The image in bottom panel depicts retention of GFP expression in spheroids formed by both cell types. The top panel also shows the percentage of cells initially plated that formed spheroids from three independent experiments. (N = 3, standard deviation reported) (D) The size of non-adherent spheroids derived from bulge and SHG McSCs when cultured in NCSC medium as determined at days 2, 4, 6 and 8. (*P ≤ 0.01, **P Value ≤ 0.05 by ANOVA; N = 5). (E) Expression of neural crest lineage markers Gfap, α-Sma, Tuj1, Krt15 among CD34+GFP+ (top panel) and CD34-GFP+ (bottom panel) McSCs following adherent culture in neural crest differentiation medium. Scale bars: 50 μm. (F) Expression of melanocyte lineage marker Tyrp1 among CD34+GFP+ (top panel) and CD34-GFP+ (bottom panel) McSCs following adherent culture in melanocyte differentiation medium. In right two panels, brightfield images show pigmented melanocytes among cultured CD34+GFP+ (top) and CD34-GFP+ (bottom) McSCs. Scale bars: 50 μm. (G) Quantification of neural crest-derived cell and melanocyte marker expression frequency after cells were cultured in either neural crest differentiation (left) or melanocyte differentiation (right) condition. For this experiment, all cells in each well were counted and this graph is a representative of 3 independent experiments.

The previous experiments demonstrated that both CD34+ and CD34- *Dct-*H2BGFP cells are McSCs, while exhibiting differences in their expression of melanogenic genes and their efficiency of HF pigmentary unit reconstitution. To characterize differences between CD34+ bulge McSCs and CD34- SHG McSCs more completely, we performed RNA-seq and analyzed the gene expression profiles of telogen-stage CD34+ and CD34- McSCs. Using an absolute log_2_-fold ≥ 1 cutoff, differential gene expression analysis of the RNA-seq reads obtained from these cells demonstrated an expected upregulation of *Cd34*, a bulge marker, in CD34+GFP+ cells and *Cdh3*, a SHG marker, in CD34-GFP+ cells. Global hierarchical clustering and volcano plot analysis showed differential expression of 3373 genes between bulge and SHG McSCs (S6A Fig, S2 and S4 Tables). Ingenuity pathway analysis (IPA) showed that ‘Axonal Guidance Signaling’ and two embryonic stem cell pluripotency categories were overrepresented in genes more highly expressed in CD34+ McSCs, whereas representation in the 'Melanocyte Development and Pigmentation Signaling’ cetagory was more equally weighted (S7 Fig and S3 Table), suggesting a differentiation state difference between the cell subsets. Since melanocytes are derived from the neural crest, we selected candidate neural crest stem cell-related genes from the literature to investigate their gene expression differences further by inspection and in comparison with known melanocyte development and differentiation genes. We found that CD34- McSCs expressed higher levels of melanogenic genes such as *Mitf*, *Tyr*, *Tyrp1*, *Pmel*, *Pax3*, *Mc1r*, *Erbb3*, *Sox10*, *Melan-A*, and *Slc45a2*, consistent with the higher expression of *Tyr*, *Tyrp1*, *Pmel17*, and *Mitf* determined by qRT-PCR (Fig 4B). In contrast, CD34+ McSCs expressed higher levels of neural crest stem cell markers *Nr2f2*, *Nr2f1* [19], *Ngfr* (*p75*) [20], *Twist1*, *Twist2*, *Snai1* [21], *Sox9* [22], *EdnrA* [23], *Gli1* [24], *Bmp2*, *Bmp4*, and *Bmp7* [25, 26]. These subsets of cell type-characteristic genes organize into distinct hierarchical clusters (Fig 4A). Quantitative RT-PCR analysis of expression of select genes in CD34+ and CD34- McSCs validated the RNA-Seq results (S6B and S6C Fig).

In a prior study, skin-derived cells expressing neural crest cell markers p75 and Sox10 grew as spheroids in culture under non-adherent conditions. These cells were reported to exhibit both melanocyte and glial differentiation potential [27]. Based upon this observation, we tested the ability of CD34+ and CD34- cells to grow as spheroids under non-adherent conditions in neural crest stem cell (NCSC) medium. Both populations of cells were capable of growth as spheroids, although spheroids from CD34+ McSCs were larger than those from CD34- McSCs (Fig 4C and 4D). The percentage of initially plated CD34+ and CD34- cells that formed spheroids was 0.43% and 0.33% respectively (Fig 4C). Cells growing as spheroids were placed in adherent, neural crest cell culture conditions and studied for expression of proteins characteristically expressed by distinct, neural crest-derived cell types, such as Gfap as a marker of glial cells, Tuj1 antigen (β3-tubulin) as a marker of neurons, and α–smooth muscle actin (α-Sma) as a marker of myofibroblasts [20], as well as the primitive keratin Krt15. Only adherent cells derived from CD34+ McSCs expressed this spectrum of neural crest-derived cell markers (Fig 4E). Adherent cells derived from CD34+ and CD34- McSC spheroids both showed expression of the melanocyte marker Tyrp1, although only cells derived from CD34- McSCs also revealed visible pigmentation (Fig 4F). Quantification of the expression of markers in individual cells (Fig 4G) showed that of all the neural crest-derived cell type markers expressed in cells derived from CD34+ McSCs, Gfap was the most frequently expressed. CD34+ McSCs also selectively expressed nestin, a neuronal stem cell marker, at higher levels (S10A and S10B Fig). Tyrp1 was expressed in the majority of cells cultured in melanocyte differentiation medium derived either from CD34+ or CD34- McSCs, although the percentage of Tyrp1-expressing cells was higher in adherent cells derived initially from CD34- McSCs (Fig 4G). The diversity of neural crest cell lineage markers expressed selectively by CD34+ McSCs suggested that these cells might possess the capability of functioning as either a neural crest stem cell or as a non-melanocytic neural crest-derived cell.

CD34+ McSCs also resemble skin-derived precursor cells (SKPs), or skin-derived stem cells, that have been previously isolated from mammalian dermis insofar as they are skin-derived, grow as spheroids in non-adherent culture conditions, and differentiate along multiple neural crest lineages [28–30]. Establishing whether SKP differentiation closely resembles the differentiation profile of CD34+ McSCs that we have previously demonstrated could provide insight into the relationship between SKPs and CD34+ McSCs. Given the ability of SKPs to undergo glial differentiation [28, 29], we wondered whether the differentiation profile of CD34+ McSCs, with their expression of multiple NCSC-associated genes, would most closely resemble SKPs as a stem cell isolated from adult skin or a more primitive embryonic NCSC. We compared the frequencies of distinct lineage markers expressed by CD34+ McSCs and murine SKPs, grown first as non-adherent spheroids, then differentiated in neural crest differentiation medium [31], to determine whether these cell populations are identical or merely overlap. We included a comparison with embryonic neural crest stem cells (eNCSCs) with this analysis. CD34+ McSCs expressed nestin, fibronectin, neuron-specific β-tubulin (Tuj-1), and α-Sma at similar frequencies to murine SKPs (Table 1, S8 Fig). However, CD34+ McSCs expressed p75/Ngfr at a much higher frequency than murine SKPs (86% vs. 3.5%). eNCSCs expressed p75/Ngfr at a similar high frequency (65%). Both the Schwann cell/oligodendroglial markers Gfap and CNPase were also more frequently expressed in CD34+ McSCs compared to SKPs (44% vs 3% and 31% vs. 3%, respectively). eNCSCs expressed these markers at an intermediate frequency (9% and 11%, respectively). When SKPs and CD34+ McSCs were instead differentiated in SKP medium [32], CD34+ McSCs again expressed higher levels of p75/Ngfr, Gfap, and CNPase than SKPs. SKP medium was unable to support the growth of eNCSCs (S1 Table). Although CD34+ McSCs located in the telogen bulge did not express Gfap *in vivo* (S9B Fig), expression could be observed in 34% of bulge McSCs following the onset of anagen (S9A and S9C Fig). These data support a model whereby CD34+ McSCs represent a subset of murine SKPs enriched for glial differentiation, and also mirror several defined properties of eNCSCs consistent with their profile as a neural crest-like stem cell.

**Table 1:** Comparison of neural crest lineage markers expressed by CD34+ McSCs, SKPs and eNCSCs using NCSC medium.

	eNCSCs	SKPs	CD34+ McSCs
p75/Ngfr	65.1% ± 12.6%	3.5% ± 0.3%	86% ± 2.8% *
Nestin	77.3% ± 11.6%	61.9% ± 0.8%	69% ± 7.8%
Fibronectin	68.3% ± 4.8%	64.5% ± 2.7%	85.5% ± 2.8%
Tuj-1	3.8% ± 2.8%	2.6% ± 0.7%	2.1% ± 1.8%
α-Sma	4.3% ± 3.4%	1% ± 0.4%	5.2% ± 0.9%
Gfap	9.4% ± 1.7%	3.1% ± 0.9%	43.6% ± 8% *
CNPase	11.3% ± 0.3%	2.6% ± 0.9%	31.4% ± 4.4% *

* Statistically significant differences between CD34+ McSCs and SKPs.

### *De novo* myelination of myelin-deficient, *Shiverer* neurons by CD34+ McSCs

**Fig 5:**
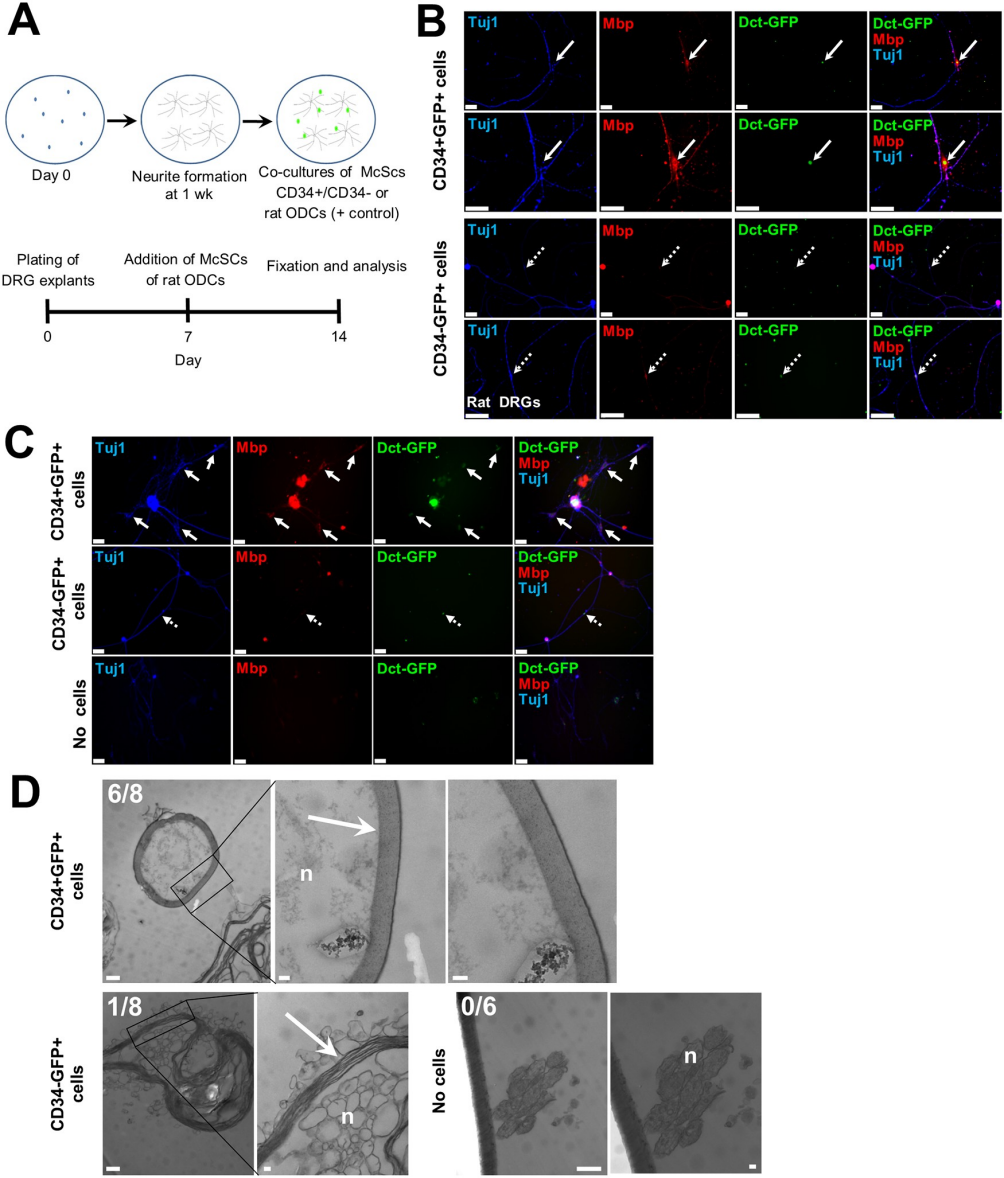
Myelination properties of CD34+ bulge McSCs. (A) Schematic of *in vitro* DRG co-culture system: CD34+, CD34- McSCs or rat ODCs and DRG cells (isolated from rat E17 or P5 *shi/shi* mice) were co-cultured for one week and screened for myelination of axons by immunofluorescence and by EM. Bottom panel shows a timeline of culturing DRG explants at Day 0, addition of McSCs (experimental) and rat ODCs (positive control) at Day 7 and fixation of cells and analysis on day 14. (B & C) Co-cultures of CD34+ or CD34- McSCs and rat eDRGs (B), or neonatal *shi/shi* DRGs (C). Arrows point to GFP-expressing cells, with solid arrow representing CD34+ bulge McSCs and dotted arrow representing CD34- SHG McSCs. In (B), second and fourth rows represents high magnification images of first and third row images. Scale bars: 50 μm. (D) Electron-dense myelin sheath formation around *shi/shi* neurites when co-cultured with CD34+ or CD34- McSCs or no added cells using EM. In the right panels in either CD34+ or CD34- McSCs co-cultured with *shi/shi* DRGs, high magnification images of the region marked with black box are shown. In the no added cells sample images with *shi/shi* DRGs, right image is a high magnification of the left image. Arrow points to the dense myelin sheath and “n” indicates neurites. Scale bars: 500 nm (non-magnified images) and 100 nm (high magnified images).

To determine whether CD34+ McSCs could recapitulate an activity of a neural crest-derived cell type other than a melanocyte, we adapted a culture system, previously used to study the ability of oligodendroglial cells (ODCs) to myelinate neurons *in vitro* [33], to assess their ability to function as glia. We isolated dorsal root ganglia (DRG) from either wild-type or C3Fe.SWV-*Mbp*^*shi/shi*^/J (*Shiverer*, *shi/shi*) mice. Mice of the *shi/shi* genotype lack myelin basic protein (Mbp) and develop a “shivering” phenotype, or tremor, eventually dying between 3–4 months of age. CD34+ and CD34- McSCs, and rat ODCs as positive controls, were co-cultured with neurites extending from wild-type or *shi/shi* DRGs and studied for their ability to express Mbp, a marker of oligodendrocytes [34] and Schwann cells [35] in a neuronal distribution (Fig 5A).

In co-cultures with rat DRGs, CD34+ McSCs selectively exhibited Mbp expression in their vicinity in a neuronal pattern (Fig 5B), similar to the pattern of Mbp expression observed with rat ODGs on *shi/shi* neurites as a positive control (S10E Fig). Furthermore, CD34+ McSCs also selectively exhibited the ability to express Mbp along *shi/shi* neurites (Fig 5C), compared with CD34- McSCs or no added cells. DRGs isolated from *shi/shi* pups for co-cultures were from homozygous mutant *shi/shi* mice as confirmed by genotyping (S10C Fig). This result suggested that CD34+ bulge McSCs selectively possess the ability to generate a *de novo* myelin sheath. To determine the ability of CD34+ McSCs to generate compact myelin indicative of functional myelination, co-cultures of CD34+ and CD34- McSCs with *shi/shi* DRGs were again initiated, with resulting cultures examined using electron microscopy (EM) for evidence of compact myelin surrounding neurites in the vicinity of McSC cell bodies (S10D Fig). EM analysis of co-cultures of CD34+ McSCs revealed evidence of compact myelin in 6/8 culture sections. In contrast, a loose myelin sheath was detected in only 1/8 CD34- McSC co-culture sections, and no myelin sheath was observed surrounding *shi/shi* neurites when no McSCs were added (Fig 5D).

**Fig 6:**
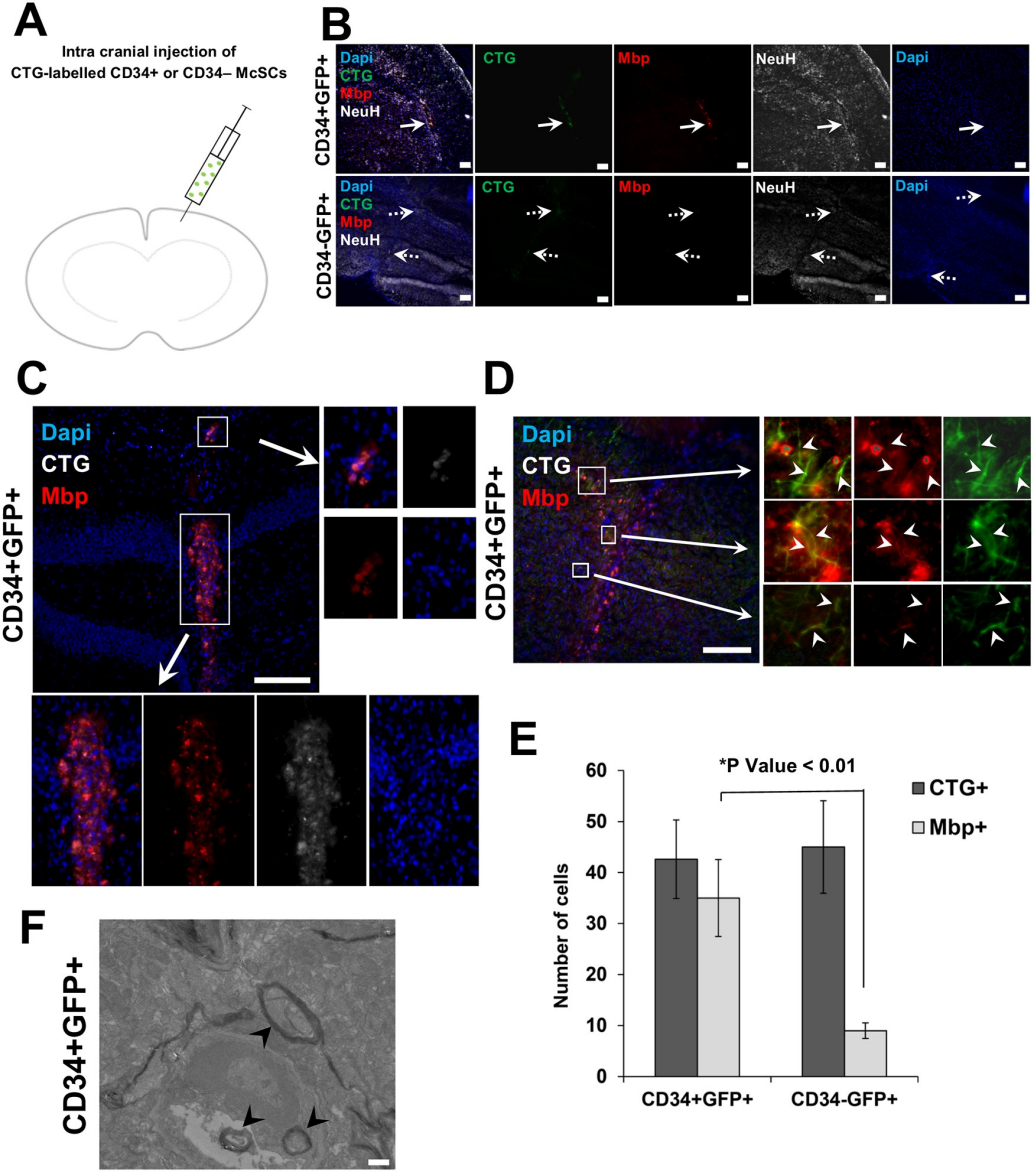
*In vivo* glial differentiation potential of CD34+ bulge McSCs. (A) Schematic diagram depicting transplantation of CD34+ or CD34- McSCs, after labelling with fluorescent CTG dye, intracranially into *shi/shi* mouse brain. At the end of the experiment, *shi/shi* mice brains were assessed for the presence of CTG cells at 2 weeks post-injection. (B) Cranial sections of *shi/shi* brains transplanted with CD34+ or CD34- McSCs show co-localization of Mbp expression in CTG-labelled CD34+ McSCs (solid arrows); co-localization is absent following transplantation of CTG-labelled CD34- McSCs (dotted arrows). (C) Cranial section of *shi/shi* brain transplanted with CD34+ McSCs shows individual CTG-labelled CD34+ McSCs co-expressing Mbp (top and bottom inset boxes). (D) A section of myelin-deficient *shi*/*shi* mice brain receiving CD34+ McSCs depicts co-localization of Mbp with neurofilament-H stained neurons in the surrounding vicinity of transplanted CTG-labelled CD34+ McSCs (arrowhead pointing within inset boxes). Scale bars: 100 μm. (E) Quantification of co-localization of Mbp expression in CTG-labelled CD34+ McSCs in cranial sections of *shi/shi* brains transplanted with CD34+ or CD34- McSCs. (*P ≤ 0.01 analyzed by ANOVA; N = 5) (F) Representative example of dense myelin sheath analyzed by transmission electron microscopy, surrounding neuron of CD34+ McSC-transplanted *shi/shi* brain (arrowhead). Scale bars: 500 nm.

To determine whether the ability of CD34+ McSCs to express Mbp was retained *in vivo*, we transplanted fluorescent dye-labelled CD34+ and CD34- McSCs into the vitreal space of *shi/shi* eyes (S11A Fig). The immunofluorescence signal of endogenous Mbp in WT eye was used as a positive control (S11B Fig). The transplanted *shi/shi* eye sections were screened for fluorescent dye-labelled McSCs. To identify the injection site histologically, we found the disrupted retinal pigmented epithelium (RPE) layer corresponding to the injection, (S11C Fig, right panels), and focused on sections from this region to identify transferred, CTG-labelled CD34+ and CD34- McSCs. In 2/2 *shi/shi* eyes receiving CD34+ McSCs, CTG-labelled cells co-expressing Mbp could be observed (S11C Fig, top row). However, in 2/2 *shi/shi* mice receiving CD34- McSCs we observed that CTG-labelled, CD34- McSCs do not co-express Mbp (S11C Fig, middle row). In a potentially more relevant neurodegenerative model, CD34+ and CD34- McSCs were cultured as non-adherent spheroids and introduced intracranially into the brain tissue of approximately 6–8 week-old *shi/shi* mice (n = 5, Fig 6A). Significant foci of Mbp expression were principally detected in brains injected with CD34+ McSCs, at the site of CTG vital dye-labeled CD34+ McSCs (Fig 6B and 6C). Quantification of co-localization of the CTG vital dye-labeled cells with Mbp showed that co-localization was observed in 82% of CTG+ cells following CD34+ McSC injection, versus 19% of CTG+ cells following CD34- McSC injection (p<0.01, Fig 6E). *Shi/shi* brain that was not injected with cells as a negative control showed no Mbp signal (S12A Fig). In addition, groups of CTG-labeled cells could be found in regions of the central (Fig 6C) and caudal brain, following injection into a rostral site, perhaps indicating some ability of injected cells to move or migrate following their introduction. Mbp expression colocalizes with neurofilament H (NeuH), a neuronal marker [36], in these foci (Fig 6D). EM analysis revealed evidence of dense myelin sheath surrounding neurons of CD34+ McSC-transplanted *shi/shi* brain (Fig 6F and S12C Fig) similar to observations in the EM positive control (S12D Fig) from Mbp-replete wild type mice (S12B Fig). All mice injected with CD34+ or CD34- McSCs survived the initial procedure, although in these experiments when the mice were euthanized 2 weeks following injection, it was not possible to determine whether CD34+ McSCs extended the normal lifespan of *shi/shi* mice.

## Discussion

Two distinct McSC populations, defined by CD34 expression, in the bulge and SHG of telogen HFs were identified and characterized using *Dct-*H2BGFP mice. Candidate gene expression analysis, melanocyte differentiation assays, enhanced efficiency of *in vivo* reconstitution of the HF pigmentary unit, and comprehensive RNA-Seq profiling results showed that CD34- SHG McSCs express melanogenic genes more highly and represent McSCs at a more advanced state of melanocyte differentiation in comparison to bulge McSCs, thereby indicating bulge and SHG McSCs are functionally distinct. Bulge CD34+ McSCs express an array of neural crest-derived cell markers, including the glial marker Gfap, and undergo glial differentiation in culture. In co-culture with DRG neurites and in murine brain, they expressed Mbp in a neuronal pattern similar to primary oligodendroglial cells and form a dense myelin sheath surrounding the neuronal axon. These data indicate that CD34+ McSCs are not exclusively committed to terminal melanocytic differentiation, readily producing myelin in a neural environment, a property normally associated with myelinating Schwann cells or oligodendroglial cells of the glial lineage. This could indicate that CD34+ McSCs contain glial cell precursors in addition to melanocyte precursors, or bipotent or multipotent progenitor cells derived from neural crest. Single-cell analysis will be required to understand further the heterogeneity and developmental potential of CD34+ McSCs.

Our data are consistent with the description of activities of other skin-derived stem cells but identify a specific cell with neural crest stem cell properties. These include studies which found evidence of p75/Sox10+ neural crest-like cells in adult murine skin HFs [27] and nestin-GFP expressing cells isolated from the HF bulge, which also exhibit the ability to express markers of multiple neural crest lineages [37]. The pluripotent, nestin-expressing GFP cells isolated from CD34+ HF bulge, which transdifferentiate largely into Schwann cells, were transplanted into the gap region of severed sciatic nerve, greatly enhancing the rate of nerve regeneration and restoring the nerve function [38]. Melanoblasts isolated from skin possess multipotency and self-renewal capabilities. The Kit-positive and CD45-negative cells isolated from embryonic melanoblast and neonatal skin HFs, when cultured on stromal cells, formed colonies containing neurons, glial cells and smooth muscle cells, together with melanocytes [39]. There is also evidence for endogenous bipotent embryonic precursors to the glial and melanocyte lineage in avians [40, 41] and in mice [41]. Our results demonstrate that a melanocyte lineage-specific cell defined by Dct, Kit, and CD34 expression in the adult HF bulge represents a neural crest-like stem cell in the skin and provide comprehensive, genome-scale characterization of its expression profile.

The skin-derived stem cells (SDSCs) isolated from mouse and human skin [28–30] exhibit glial cell differentiation and express Mbp *in vitro* similar to CD34+ McSCs isolated from HFs of *Dct*-H2BGFP mice. Mouse and human SDSCs from mammalian dermis grow as spheroids in non-adherent culture, express nestin, and possess multipotency to generate neural and mesodermal progeny [29]. When transplanted into *shi/shi* mice, SDSCs from human- and mouse-derived Schwann cells repaired peripheral nerve damage and myelinated neuronal axons of the dysmyelinated brain [42]. However, the cell of origin of these skin-derived cells with unique regenerative properties has not yet been identified specifically. CD34+ McSCs may represent a highly-specific subset of SDSCs. Although desert hedgehog (*Dhh*) is selectively expressed by myelinating Schwann cells [43] it was not differentially expressed between the two McSC subsets. This may reflect the fact that its expression is either only activated in the peripheral environment, or that other factors may be responsible for hedgehog pathway activity in CD34+ McSCs as suggested by their higher expression of Gli1 (Fig 4B). Mbp expression was higher in CD34+ McSCs, although the higher level of expression was not statistically significant in the RNA-Seq profiles (S4 Table). Mbp could be detected both *in vitro* (Fig 5C) and *in vivo* (Fig 6B) at low levels in cells derived from CD34- McSCs, although expression in these environments was more robust and more clearly in a neuronal distribution when derived from CD34+ McSCs. Nonetheless, robust myelination was correlated strongly with CD34+ McSCs *in vitro* (Fig 5D) and *in vivo* (Fig 6E and S12C Fig). Environmental cues are likely to be extremely important for determining the terminal differentiation characteristics of CD34+ and CD34- McSCs in a manner that cannot be readily predicted from their extant gene expression profile differences. Our ability to define with specific cellular markers the identity of these cells, not relying simply upon their growth characteristics and functional properties, will facilitate their comparison with McSCs in other species and enhance efforts to define highly-specific human skin-derived stem cell subsets with unique regenerative properties that can be leveraged for these therapeutic purposes.

The unique properties of CD34+ McSCs in the bulge, with their ability to myelinate neurons and express markers of non-melanocytic lineages, may be relevant to their role in this HF region where *Shh*-expressing neurons approach the HF [44] and where the arrector pili muscle attaches [45]. CD34+ McSCs may provide regenerative support to maintain the function of these extrafollicular tissues in the HF environment. Although McSCs have previously been described both in the bulge and the subbulge/SHG regions [2, 15, 46, 47], some recent studies have emphasized selectively the contribution of the subbulge/SHG subset either in the follicular onset of proliferation and differentiation [7] or in UV-induced emergence from quiescence and melanomagenesis [48]. These and other reports on McSCs have not distinguished between the bulge and subbulge/SHG subsets and their distinct functional properties. Given the heterogeneity that has been described inherently in melanoma cells and their treatment-resistant subpopulations [49], recognition of the variation in McSC phenotypes may be especially important to further define the contribution of normal McSCs to cancer development.

## Materials and methods

### Ethics statement

All mouse procedures were approved by the Institutional Animal Care and Use Committee protocol of the University of Maryland School of Medicine (IACUC protocols 0917008, 1014005).

### Mice

*Dct-*H2BGFP mice that were generated by intercrossing *Dct*-tTA *(*Hprt^tm1[Dct-tTA-SV40p(A)]Hyk^) and TRE-H2BGFP (Tg(tetO-HIST1H2BJ/GFP)47Efu) mice (gift of E. Fuchs) to the homozygous state were described previously [15]. C57BL/6-Mitf^*Mi-wh/Mi-wh*^ homozygotes were obtained originally from Dr. Lynn Lamoureux, Texas A&M College of Veterinary Medicine, and maintained on the C57BL/6 background by serial backcross. C3Fe.SWV-Mbpshi/shi/J (shiverer, *shi/shi*; RRID: IMSR_JAX:001428); athymic Foxn1^nu^ (*nu/nu*; nude); (Wnt1-Cre)2Sor (*Wnt1-Cre*); Tg(Tyr-Cre/ERT2)13Bos, transgene insertion 13 (*Tyr-CreER*); and Gt(ROSA)26Sor^tm14(CAT-tdTomato)Hze^ (R26-tdTomato) mice were purchased from The Jackson Laboratories.

### Isolation of HF McSCs from subepidermal skin and fluorescence activated cell sorting (FACS)

Dorsal skin samples were obtained from transgenic mice at the indicated ages immediately following euthanasia and fat was removed from the dermis using fine forceps. Defatted skin was incubated in 0.5% of trypsin (USB) dissolved in PBS at 37°C for 30 min. Epidermis was peeled away from the dermis following incubation, and the remaining dermis including HFs was cut into small pieces. The dermal fragments were incubated in digestion medium containing 0.2 mg/mL Liberase Thermolysin low (Roche) in a 37°C water bath for 45 to 60 min. The digested dermal mixture was added into PBS containing 0.05% DNase (Sigma) and 5% FBS. Single cells were extracted from the dermis by repeated plunging with a 60 mL syringe followed by filtration through 40 μm nylon mesh (BD Falcon). The dermal cell suspension is prepared in 5% FBS/PBS prior to incubation with respective antibodies and FACS. This procedure was done under aseptic conditions [15].

To separate the hair follicle bulge/LPP and SHG cells of *Dct-*H2BGFP bitransgenic mice using FACS, the cell suspension was incubated with Alexa 647-conjugated anti-CD34 antibody in for 30 min at 4°C. 7-AAD was added to the CD34-labeled dermal cell suspension in 5% FBS/PBS to discriminate between live and dead cells, and cell sorting was performed using BD FACSAria1 (Becton-Dickenson) on a 100 μm nozzle at 20 psi low sheath pressure. Sorted cells were counted and used either for primary cultures or for quantitative realtime PCR (qRT-PCR) by extracting RNA from respective cell populations.

### *In vitro* cell culture

For melanocyte differentiation medium (MDM) culture, 3000 cells of CD34+ or CD34- McSCs at equal density were plated in 24 well plates with MDM containing 5% FBS, stem cell factor (SCF; 50 ng/mL; Peprotech), endothelin-3 (20 nM; Sigma), basic fibroblast growth factor (bFGF; 2.5 ng/mL; R&D Systems), α-melanocyte stimulating hormone (α-MSH; 100 nM; Sigma), phosphoethanolamine (1 M; Sigma), ethanolamine (10 M; Sigma), insulin (1 mg/mL; Sigma) and 1% Penicillin/streptomycin in RPMI 1640 medium [50].

For culturing McSCs as spheroids in non-adherent culture conditions, 4000 cells of CD34+ or CD34- McSCs at equal density were placed in ultra-low attachment 24-well plates, with neural crest stem cell (NCSC) medium as described previously [31, 51] containing DMEM (low glucose) (Gibco Life technologies), 30% neurobasal medium (Gibco Life technologies), 15% chick embryo extract (US Biological), 2% B27 supplement (Gibco Life technologies), 1% N2 supplement (Gibco Life technologies), 117 nM retinoic Acid (Sigma), 50 μM β-mercaptoethanol (Sigma), 20 ng/mL insulin-like growth factor (IGF; Sigma), 20 ng/mL bFGF (R & D System) and 1% penicillin/streptomycin (Gibco Life technologies). For McSC cultures in adherent conditions to evaluate neural crest lineage marker expression, cells were cultured in 30 μg/mL fibronectin-coated (Corning) 8-well chamber slides (Lab-Tek II, Nunc) in neural crest differentiation medium, which is NCSC medium that instead contains 1% chick embryo extract and 10 ng/mL bFGF, for 8 days as described previously [31].

Rat glial precursor cells (Gibco Life technologies) were cultured on Poly-D-Lysine (Sigma) coated plates in glial precursor cell growth (GPCG) medium containing KnockOut DMEM/F-12, 2 mM Glutamax supplement, 2% StemPro NSC SFM supplement (Gibco Life technologies), 20 ng/mL bFGF, 20 ng/mL EGF (Promega), and 10 ng/mL PDGF-AA (eBioscience) as per the manufacturer’s descriptions. To spontaneously induce differentiation of glial precursor cells into mature oligodendroglial cells (ODCs), cells were cultured on Laminin (Sigma) and Poly-D-Lysine coated plates in glial differentiation culture medium, defined as GPCG but without PDGF-AA and bFGF.

For the comparison of CD34+ McSCs with murine SKPs and eNCSCs, these cell types were grown as spheroids in NCSC medium or SKP medium for 7 days in ultra-low attachment plates. The SKP medium consists of DMEM/F12 (Gibco Life technologies), B27 supplement, 40 ng/mL FGF, 20 ng/ml EGF and penicillin/streptomycin as described previously [32]. For CD34+ McSCs, murine SKPs, and eNCSC adherent cultures to evaluate neural crest lineage marker expression, cells were cultured in neural crest differentiation medium as described above or SKP differentiation conditions for a week. SKP differentiation medium consists of DMEM/F12, B27 supplement, 3% FBS and penicillin/streptomycin as described previously [32]. For SKP studies, cells were isolated by preparing a dermal single cell suspension from skin of C57BL6 mice at P56 as described above. For the isolation of eNCSCs, neural tubes were isolated from *Dct*-H2BGFP embryos at E9.5 and then cultured in NCSC medium. After 24 hours of incubation at 37°C, eNCSCs migrated away from the cultured neural tube and were analyzed experimentally [51].

### Dorsal root ganglion (DRG) co-cultures

Isolation and culture of DRGs from C3Fe.SWV-*Mbp*^*shi/shi*^/J (shiverer, *shi/shi*) pups was performed as described previously [52]. *Shi/shi* pups (P5 to P8) were euthanized according to institutional guidelines and the spine extracted. Excess muscle and bone from the spine was trimmed away and the spine placed in a Petri dish ventral side up. Using dissection scissors the spinal column was cut along the midline starting caudally in a longitudinal fashion. The spinal column was gently opened by two pairs of forceps and the spinal cord exposed. Using fine-tipped forceps the DRGs, found beneath and lateral to the spinal cord, were removed and transferred to ice cold Hank’s buffered salt solution (HBSS) in a new Petri dish. DRGs were transferred to a 1.5 mL centrifuge tube containing 500 μl of ice-cold HBSS and were pelleted by spinning at 1200 rpm for 5 min at 4°C. The supernatant was discarded and a 500 μL solution of pre-warmed DRG papain solution was added followed by incubation at 37°C for 10 min. Following another light spin, the supernatant was discarded and 500 μL of pre-warmed Collagenase A solution was added followed by incubation at 37°C for 10 min. Following another light spin and removal of supernatant, DRGs were washed twice with 1mL of DRGN media (DMEM containing 10% FBS). Finally, DRGs were dissociated by triturating them with a BSA-coated glass Pasteur pipette. After dissociation, the suspension was passed through a 40 μm filter into a sterile Petri dish containing 7 mL of DRGN media. This suspension was incubated at 8.5% CO_2_ for 1h 15 min. At this stage many contaminating cells including fibroblast and glial cells strongly adhered to the Petri dish, enriching cell suspension for DRGs. The cell suspension containing DRGs was collected by pelleting and cultured in 10 μg/mL Laminin (and 30 μg/mL Poly-D-Lysine coated 24-well plates in DRGN media at 8.5% CO_2_, 37² C overnight. The next day, DRGN media was replaced with OL media (DMEM with 2% B27 supplement, 1% N2 Supplement, 1x glutamine, 0.5% FBS and 1% penicillin/streptomycin) with 10 μM 5’-Fluoro-deoxyuridine (FuDR; Sigma) to prevent the proliferation of contaminating fibroblasts and glial cells. On Day 5, full media was changed with OL media (without FuDR) and by Day 7, the DRGs had formed an extensive neurite bed, ready for co-culture.

For DRG co-cultures, CD34+ or CD34- McSCs isolated from *Dct*-H2BGFP mouse skin or rat oligodendroglial cells (ODC; Gibco Life technologies) were seeded onto the dense neuronal bed generated either by *shi/shi* or rat embryonic DRGs. For co-cultures of CD34+ or CD34- McSCs and *shi/shi* or rat embryonic DRGs, cells were cultured in neural crest differentiation medium containing 10μM ascorbic acid to induce myelination. For co-cultures of rat ODCs and *shi/shi* DRGs, cells were cultured in glial cell differentiation medium. After one week of co-culture, cells were fixed and examined for Mbp expression by immunofluorescence and myelin sheath formation by electron microscopy.

### Intracranial and Intraocular transplantation of CD34+ and CD34- McSCs

FACS-sorted CD34+ or CD34- McSCs from *Dct*-H2BGFP mouse skin were grown as spheroids in NCSC medium for a week. After dissociating spheroids into a single cell suspension, CD34+ and CD34- McSCs were labelled with a fluorescent dye, CellTracker Green (CTG) (Molecular Probes) as per the manufacturer’s recommendations. For intracranial injections, an incision of the skin was cut over the lateral skull and a 2 mm hole was drilled in the skull over the hippocampus and the striatum of *shi*/*shi* brain. Intracranial injections [53] of CTG-labelled CD34+ or CD34- McSCs in 5 μL PBS with a Hamilton syringe were performed into the unilateral cranium of anesthetized 6–8 week old *shi/shi* mice. After the injections, the skin is sutured with sterile nylon monofilament suture. Intraocular injections [53, 54] of CTG-labelled CD34+ or CD34- McSCs in 5 μL PBS were performed into the vitreal space of anesthetized 8 week old *shi/shi* mice. These procedures were performed under the auspices of an approved Institutional Animal Care and Use Committee protocol of the University of Maryland School of Medicine. Control *shi/shi* mice were injected with 5 μL PBS only.

Additional information is available in the S1 Text: **Supplemental materials and methods**.

## Supporting information

S1 Fig *Dct*-H2BGFP-expressing melanocyte precursors in telogen HFs of mouse tail skin and separation of bulge and SHG McSCs. (A & B) The *Dct*-H2BGFP-expressing McSCs in CD34- SHG region (arrowheads, A) and P-cad+ SHG region (arrowheads, B) of telogen HFs of mouse tail skin sections. In (A) arrows demonstrate *Dct*-H2BGFP-expressing interfollicular epidermal melanocytes in tail skin of *Dct*-H2BGFP mouse. Scale bars: 50 μm. (C & D) P56 whole mount HFs of mouse tail epidermis demonstrating *Dct*-H2BGFP-expressing McSCs in CD34+ bulge/LPP (arrowheads, C) and P-cad+ SHG regions (arrowheads, D). Scale bars: 50 μm. (E) Representative FACS sorting schemes are shown for isolation of bulge/LPP and SHG melanocyte precursors based on GFP and CD34 expression from *Dct*-H2BGFP and wild type mouse skin HFs. DP = Double positive, SP = Single positive and DN = Double negative. (F) Reanalysis for the purity of CD34+GFP+ (bulge/LPP) and CD34-GFP+ (SHG) FACS sorted melanocyte precursors. Reanalysis of sorted cell populations showed >91% CD34+GFP+ and 97% CD34-GFP+ cells retained respective cell markers. (G) RNA quality of extracts from sorted CD34+GFP+ and CD34-GFP+ cells. Bioanalyzer analysis of the total RNA extracted from the FACS-sorted GFP+ melanocytes showed high quality RNA with intact 18S and 28S ribosomal RNA bands.
(TIF)

S2 Fig Co-localization of CD34 and *Dct*-H2BGFP-expressing melanocyte precursors in different HF cycle stages of *Dct*-H2BGFP mice. The *Dct*-H2BGFP-expressing McSCs show co-localization with CD34 in different HF cycle stages of *Dct*-H2BGFP mice. First anagen (P8; A), first telogen (P21; B), second anagen (P30; C), second telogen (P56; D) and third anagen (P70; E). Scale bars: (A, C, E 100 μm) (B & D 50 μm). (A and B) Arrowheads in the inset images depict the GFP+ nucleus and the surrounding CD34 expression in the individual channel and composite images. (F) Quantification of *Dct*-H2BGFP-expressing McSCs co-expressing CD34. For this experiment only upper and lower ORS *Dct*-H2BGFP-expressing McSCs were counted and mature bulb *Dct*-H2BGFP-expressing melanocytes were eliminated.
(TIF)

S3 Fig Co-localization of Tyr and Tyrp1 in *Dct*-H2BGFP-expressing mature HF melanocytes of *Dct*-H2BGFP mice. The *Dct*-H2BGFP-expressing mature melanocytes show co-localization with Tyr (A) and Tyrp1 (B) in anagen HFs of *Dct*-H2BGFP mice at P30 whereas bulge McSCs do not. Scale bars: 100 μm.
(TIF)

S4 Fig The purity of FACS sorted cells by immunofluorescence: CD34+GFP+ express Kit and CD34, whereas CD34-GFP+ cells express only Kit. To test the purity of FACS sorted cells, CD34+GFP+, CD34-GFP+, CD34+GFP- and CD34-GFP- cells, prior to any cell culture conditions, were directly cytospun onto the slide, fixed, and stained for Kit, CD34 and K14. Co-localization of KIT (A) was observed among CD34+GFP+ and CD34-GFP+ FACS sorted cells. In contrast, co-localization of CD34+ (B) was restricted to CD34+GFP+ and CD34+GFP- sorted cells and (C) K14 a HF keratinocyte marker was restricted to CD34+GFP- and CD34-GFP- sorted cells. Scale bars: 100 μm.
(TIF)

S5 Fig *In vitro* and *in vivo* melanocyte differentiation potential of bulge and SHG McSCs. (A) & (B) Supplementary information for Fig 3B. (A) The images show three different categories used to quantify differentiation potential of bulge and SHG McSCs: Round cells (top panel), dendritic cells (middle panel) and pigmented cells (bottom panel). (B) Quantification of total GFP-expressing cells of CD34+ and CD34- McSCs when cultured in melanocyte culture condition at Day 4 and Day 7. (*P ≤ 0.01, **P Value ≤ 0.05 by ANOVA) (C) Immunofluorescence staining shows identification of GFP-expressing McSCs in CD34+ bulge (arrow head) and CD34- SHG (arrow) HFs of the skin grafts receiving either CD34+GFP+ or CD34-GFP+ McSCs at 2 months post-surgery. Scale bars: 50 μm.
(TIF)

S6 Fig Differential expression of genes from RNA-Seq analysis of CD34+ and CD34- McSCs. (A) Heatmap using sample clustering shows distinct differential gene expression pattern between CD34+ and CD34- McSCs. Red represents high expression of genes and blue represents low expression of genes (upper panel). MA plot of differentially expressed genes identified in CD34+ and CD34- McSCs. Data represent individual gene responses plotted as log2 fold-change CD34+/CD34- versus mean of normalized counts. FDR <0.02 was used as a cutoff to determine significant differential gene expression between two cell types. Positive and negative change represents the up-regulated genes in CD34+ and CD34- McSCs respectively and are highlighted in red (lower panel). (B) RT-PCR results show and validate higher expression of melanogenic genes and transcription factors: *Pax3*, *Slc45a2*, *Erbb3* and *Sox10* in CD34-/SHG McSCs (*P ≤ 0.01 by ANOVA). (C) Likewise, CD34+/bulge McSCs show higher expression of neural crest stem cell markers like *Ngfr*, *Bmp7* and *Gli1* (*P ≤ 0.01 by ANOVA).
(TIF)

S7 Fig Classification of differentially expressed genes between CD34+ and CD34- McSCs into various canonical pathway categories by IPA. The figure depicts the highest 60 categories of the display that summarizes all 435 canonical pathways based on IPA of 3,220 differentially expressed genes (P value≤ 0.01) between CD34+ (bulge) and CD34-(SHG) McSCs. The orange line indicates the likelihood (-log(p-value)) that the genes in a specific category are differentially expressed. The stacked bar graphs show the percentage of genes that are upregulated in CD34+ McSCs (red), are downregulated in CD34+ (green) or have no overlap between the 2 McSC subsets (white). The selected top section of the graph highlights categories related to neural crest stem cells like ‘axonal guidance signaling’, ‘human embryonic stem cell pluripotency’, ‘role of NANOG in mammalian embryonic stem cell pluripotency’ and ‘mouse embryonic stem cell pluripotency’. In these categories, a higher number of genes is upregulated in CD34+ McSCs compared to CD34- McSCs. Similarly, the figure also shows the ‘melanocyte development and pigmentation signaling’ category where approximately half the genes are upregulated in CD34+ McSCs while the other half are upregulated in CD34- McSCs.
(TIF)

S8 Fig Comparison of cultured CD34+ McSCs with SKPs and eNCSCs. (A) CD34+ McSCs, murine SKPs and eNCSCs are grown as spheroids in NCC medium and SKP medium for 7 days. The efficiency of spheroid formation is provided at the top of each panel (N = 3). Scale bars: 100 μm. (B, C, D and E) Cells are then differentiated in neural crest differentiation medium (B and C) and SKP differentiation medium (D and E). Marker comparison is performed at early (24 hours) and late (1 week) timepoints. Immunofluorescence staining of p75, nestin and fibronectin at the early differentiation stage (B and D) and α-Sma, Tuj1, Gfap and CNPase at the late differentiation (C and E). Scale bars: 75 μm.
(TIF)

S9 Fig GFP-expressing McSCs in upper bulge region of anagen HF co-express Gfap. (A) GFP-expressing cells co-express Gfap (inset boxes) in the upper bulge region of growing anagen HFs in *Dct*-H2BGFP mice at P70. In the image, bracket depicts a distinct Gfap stained upper bulge region of elongating HF in *Dct*-H2BGFP mice. (B) In telogen HF of *Dct*-H2BGFP mice at P56, the bulge GFP-expressing cells lack Gfap expression (dotted arrow). (C) Quantification data show 34% of bulge *Dct*-H2BGFP-expressing cells reveal co-localization with Gfap at the onset of anagen in *Dct*-H2BGFP mice at P70. For this experiment, only upper and lower ORS *Dct*-H2BGFP-expressing McSCs were counted and mature bulb *Dct*-H2BGFP-expressing melanocytes were eliminated.
(TIF)

S10 Fig CD34+ bulge McSCs exhibit neuronal stem cell marker nestin and co-cultures of McSCs and *shi/shi* DRGs. Comparison of expression of nestin mRNA (A) and protein (B) among CD34+ bulge and CD34- SHG McSCs. (C) Genotyping to identify *shi/shi* pups which were further used to isolate DRGs at P5 to P8. For each of two separate experiments, an individual litter was genotyped as shown in top and bottom panel. (D) A representative image for GFP-expressing cells (CD34+ or CD34- or no cells) co-cultured with neurites generated from DRGs isolated from *shi/shi* pups. After the localization of GFP-expressing cells in their representative cultures, cells were then fixed and analyzed with EM. (E) Co-cultures of ODCs and neonatal *shi/shi* DRGs as positive control. The top row depicts Mbp expressed by ODCs (left panel) and Tuj1 expressed by *shi/shi* axonal outgrowths (center). At bottom row, high magnification images of the region marked with white box are shown; they depict Mbp deposition along a Tuj1-expressing *shi/shi* axon.
(TIF)

S11 Fig Transplantation of CD34+ McSCs in *shi/shi* mice eye. (A) Transplantation of CD34+ or CD34- McSCs, after labelling with fluorescent CTG dye, into the vitreous space of *shi/shi* mouse eye for 10 days. (B) Immunofluorescence for the endogenous expression of Mbp around the retinal layer (arrow) and ciliary body (arrow head) in wild type mouse eye. (C) Retinal sections of *shi/shi* eyes transplanted with CD34+ or CD34- McSCs show co-localization of Mbp expression in CTG-labelled CD34+ McSCs (solid arrows), whereas there is no evidence of Mbp expression in CTG-labelled CD34- McSCs (dotted arrows). Arrowhead points to the injection site in each image. The bottom panels represent a no cell-injected retinal section from control *shi/shi* mice. Scale bars: 100 μm.
(TIF)

S12 Fig Mbp expression and myelin sheath formation in brain sections of *shi/shi* and WT mice. (A) Cranial sections of *shi/shi* brains not receiving transplanted cells show lack of Mbp expression. Scale bars: 50 μm. (B) Cranial sections of wild-type brains (positive control) show high expression of Mbp and its expression was co-localized with neurofilament H (NeuH)-expressing neurons. Upper panel is a high magnification image and bottom is a low magnification image. Scale bars: 50 μm. (C) TEM image shows dense myelin sheath image of brain sections receiving CD34+GFP+ cells whereas there is lack of myelin sheath formation in brain sections of *shi/shi* mice receiving no cells. (D) TEM image shows dense myelin sheath around neurons of brain sections of wild-type mice whereas lack of myelin sheath formation in brain sections of *shi/shi* mice receiving no cells. Scale bars: 500 nm.
(TIF)

S1 Table Comparison of neural crest lineage markers expressed by CD34+ McSCs, and SKPs using SKP medium. (DOCX)

S2 Table Differentially expressed genes between CD34+ and CD34- McSCs. The attached Excel file includes the alignment statistics, differential expression (DE) summary and the corresponding data sheets. Samples used for RNA-seq for both CD34+ and CD34- McSCs are in triplicates. From 100 base pair reads, DE analysis was performed with the results filtered to include only those that met an FDR cutoff of 0.01, read count percentile (RCP) of 25%, and a log fold change (LFC) change of 1 or -1. With the RCP set at 0.25, the lower limit cutoff for DE was set to the normalized read count of 16.93. The data sheet lists all the 3,220 DE genes between the 2 McSC subsets identified using mentioned parameters.
(XLSX)

S3 Table Ingenuity Pathway Analysis (IPA) of canonical pathways between CD34+ and CD34- McSCs. The Excel file lists differentially expressed genes between CD34+ and CD34- McSCs in various canonical pathway categories according to Ingenuity Pathway Analysis (IPA) results. The table lists all canonical pathway categories, their–(log p-value), ratio, z-score, downregulation (in CD34+ McSCs compared to CD34- McSCs), no change, upregulation (in CD34+ McSCs compared to CD34- McSCs) and no overlap DE genes percentages. It also lists all the DE molecules in each canonical pathway category.
(XLS)

S4 Table Raw counts for all genes for CD34+ and CD34- McSCs that were generated by HTSeq-count. The Excel file comprises of two separate spreadsheets: (a) with all gene counts and (b) without genes with zero counts for all six samples (3 CD34+ McSC samples and 3 CD34- McSC samples).
(XLSX)

S5 Table Raw data and statistical analysis used to generate graphs. (XLSX)

S1 Text Supplemental materials and methods. (DOCX)
