# Supplementary figures and images for: CD34 defines melanocyte stem cell subpopulations with distinct regenerative properties
Source: PLoS Genet. 2019 Apr 24;15(4):e1008034. doi: 10.1371/journal.pgen.1008034 (PMC6481766; doi:10.1371/journal.pgen.1008034)

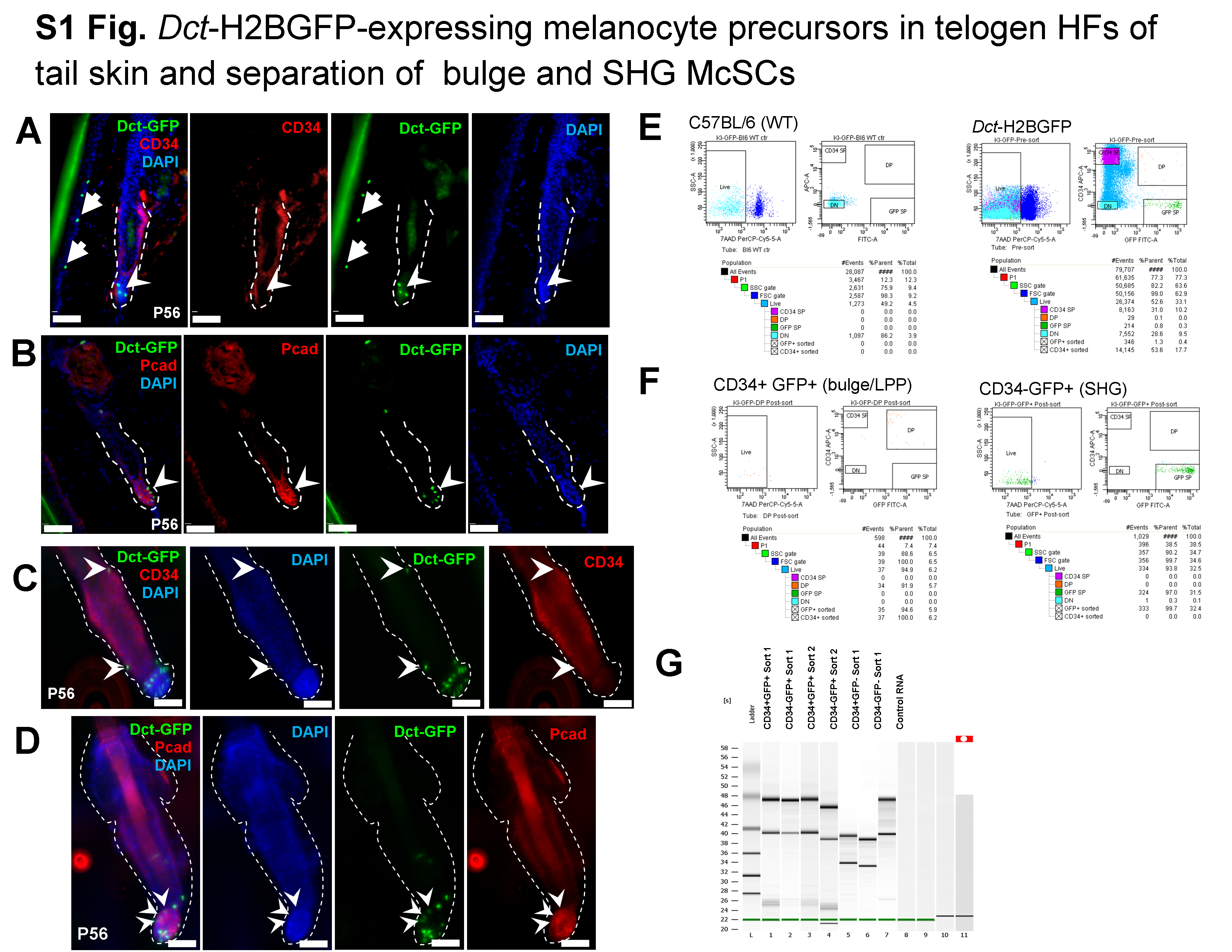

Supplement: S1 Fig — (A & B) The Dct-H2BGFP-expressing McSCs in CD34- SHG region (arrowheads, A) and P-cad+ SHG region (arrowheads, B) of telogen HFs of mouse tail skin sections. In (A) arrows demonstrate Dct-H2BGFP-expressing interfollicular epidermal melanocytes in tail skin of Dct-H2BGFP mouse. Scale bars: 50 μm. (C & D) P56 whole mount HFs of mouse tail epidermis demonstrating Dct-H2BGFP-expressing McSCs in CD34+ bulge/LPP (arrowheads, C) and P-cad+ SHG regions (arrowheads, D). Scale bars: 50 μm. (E) Representative FACS sorting schemes are shown for isolation of bulge/LPP and SHG melanocyte precursors based on GFP and CD34 expression from Dct-H2BGFP and wild type mouse skin HFs. DP = Double positive, SP = Single positive and DN = Double negative. (F) Reanalysis for the purity of CD34+GFP+ (bulge/LPP) and CD34-GFP+ (SHG) FACS sorted melanocyte precursors. Reanalysis of sorted cell populations showed >91% CD34+GFP+ and 97% CD34-GFP+ cells retained respective cell markers. (G) RNA quality of extracts from sorted CD34+GFP+ and CD34-GFP+ cells. Bioanalyzer analysis of the total RNA extracted from the FACS-sorted GFP+ melanocytes showed high quality RNA with intact 18S and 28S ribosomal RNA bands. (TIF) [file pgen.1008034.s001.tif]

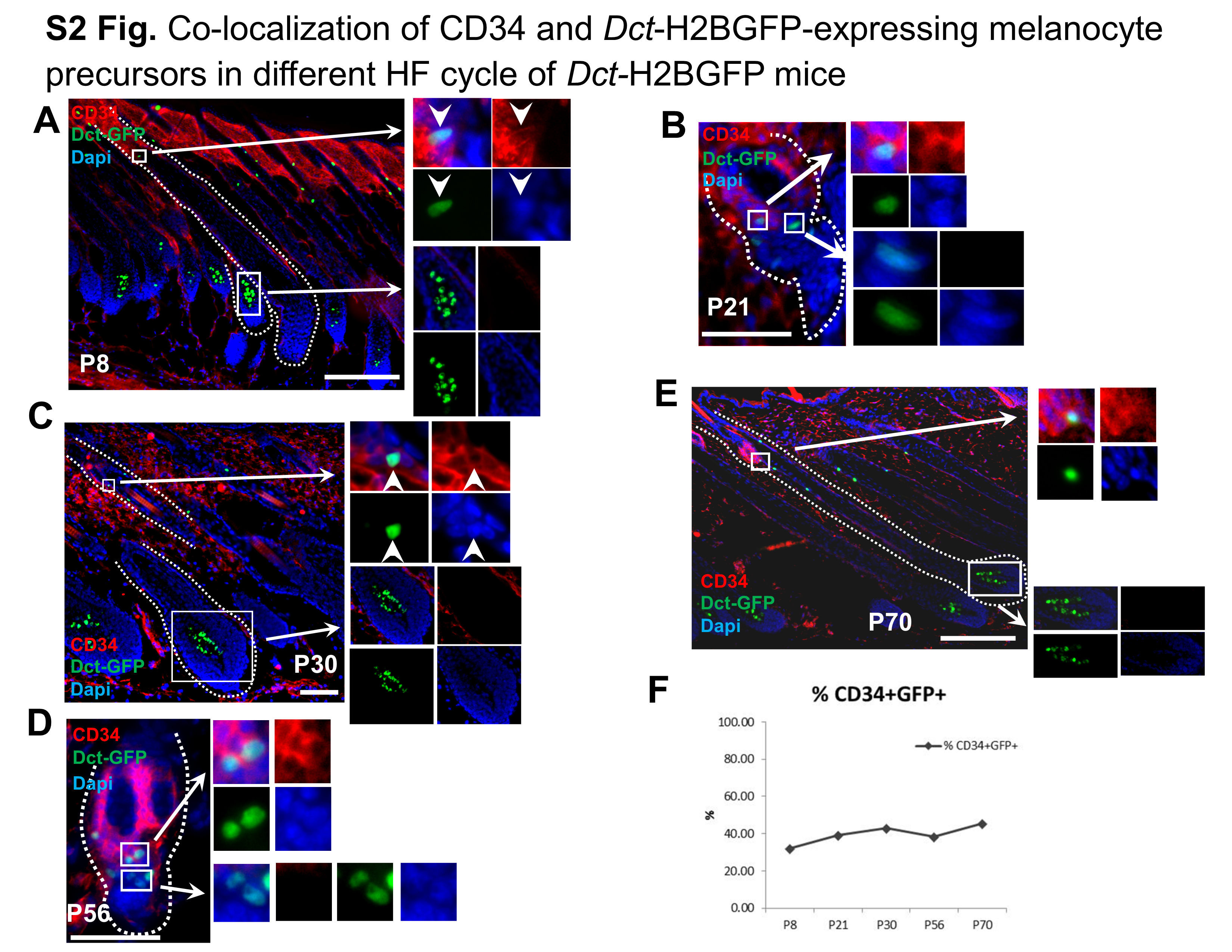

Supplement: S2 Fig — The Dct-H2BGFP-expressing McSCs show co-localization with CD34 in different HF cycle stages of Dct-H2BGFP mice. First anagen (P8; A), first telogen (P21; B), second anagen (P30; C), second telogen (P56; D) and third anagen (P70; E). Scale bars: (A, C, E 100 μm) (B & D 50 μm). (A and B) Arrowheads in the inset images depict the GFP+ nucleus and the surrounding CD34 expression in the individual channel and composite images. (F) Quantification of Dct-H2BGFP-expressing McSCs co-expressing CD34. For this experiment only upper and lower ORS Dct-H2BGFP-expressing McSCs were counted and mature bulb Dct-H2BGFP-expressing melanocytes were eliminated. (TIF) [file pgen.1008034.s002.tif]

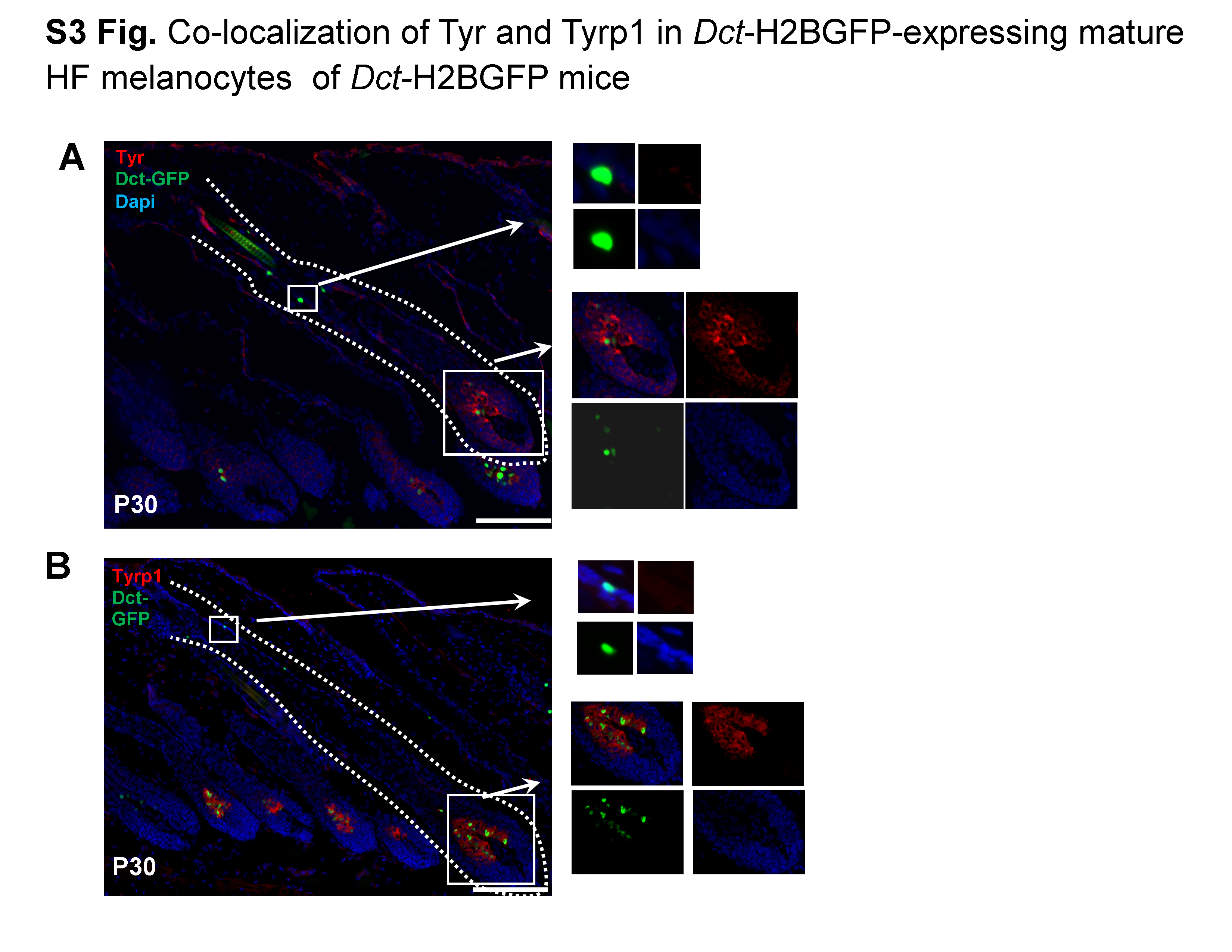

Supplement: S3 Fig — The Dct-H2BGFP-expressing mature melanocytes show co-localization with Tyr (A) and Tyrp1 (B) in anagen HFs of Dct-H2BGFP mice at P30 whereas bulge McSCs do not. Scale bars: 100 μm. (TIF) [file pgen.1008034.s003.tif]

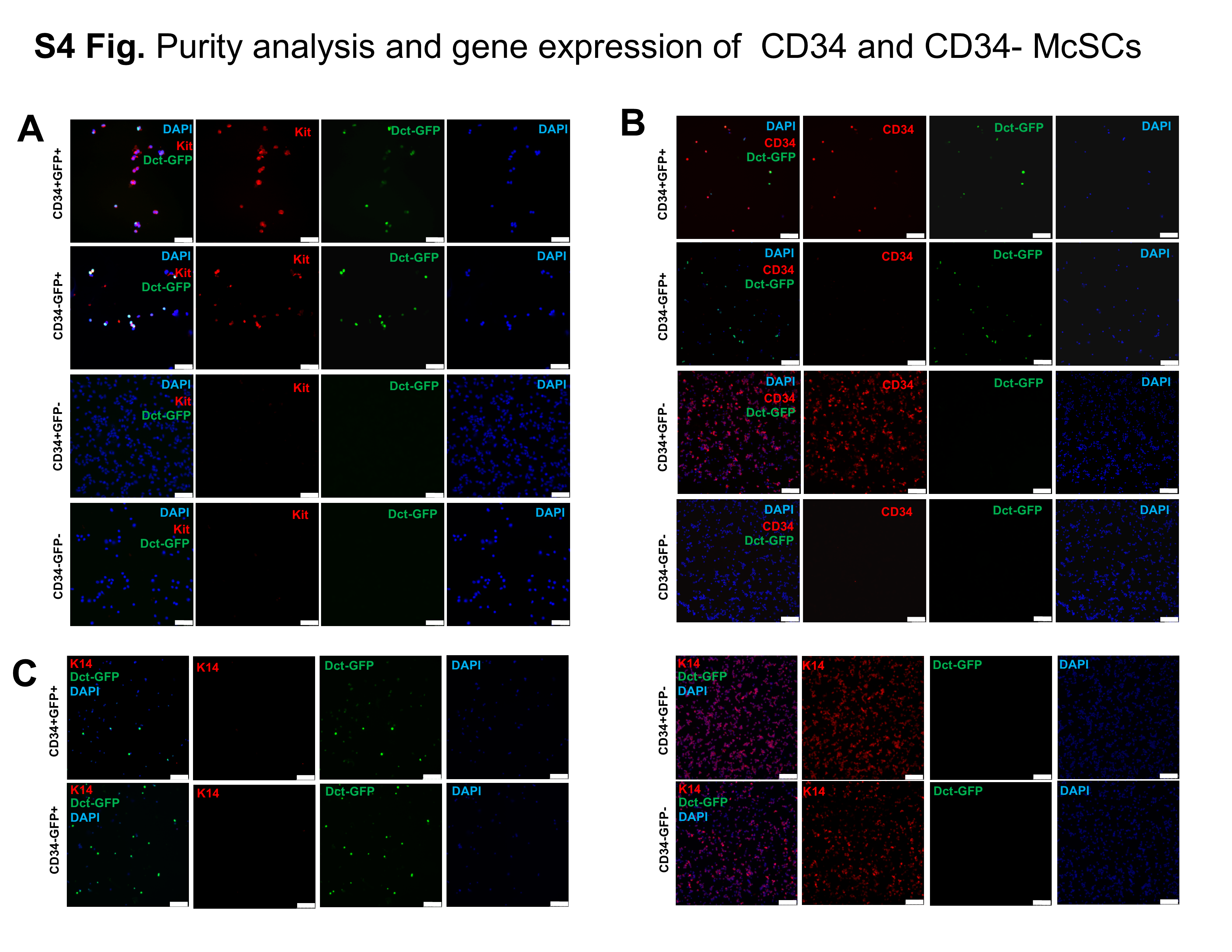

Supplement: S4 Fig — To test the purity of FACS sorted cells, CD34+GFP+, CD34-GFP+, CD34+GFP- and CD34-GFP- cells, prior to any cell culture conditions, were directly cytospun onto the slide, fixed, and stained for Kit, CD34 and K14. Co-localization of KIT (A) was observed among CD34+GFP+ and CD34-GFP+ FACS sorted cells. In contrast, co-localization of CD34+ (B) was restricted to CD34+GFP+ and CD34+GFP- sorted cells and (C) K14 a HF keratinocyte marker was restricted to CD34+GFP- and CD34-GFP- sorted cells. Scale bars: 100 μm. (TIF) [file pgen.1008034.s004.tif]

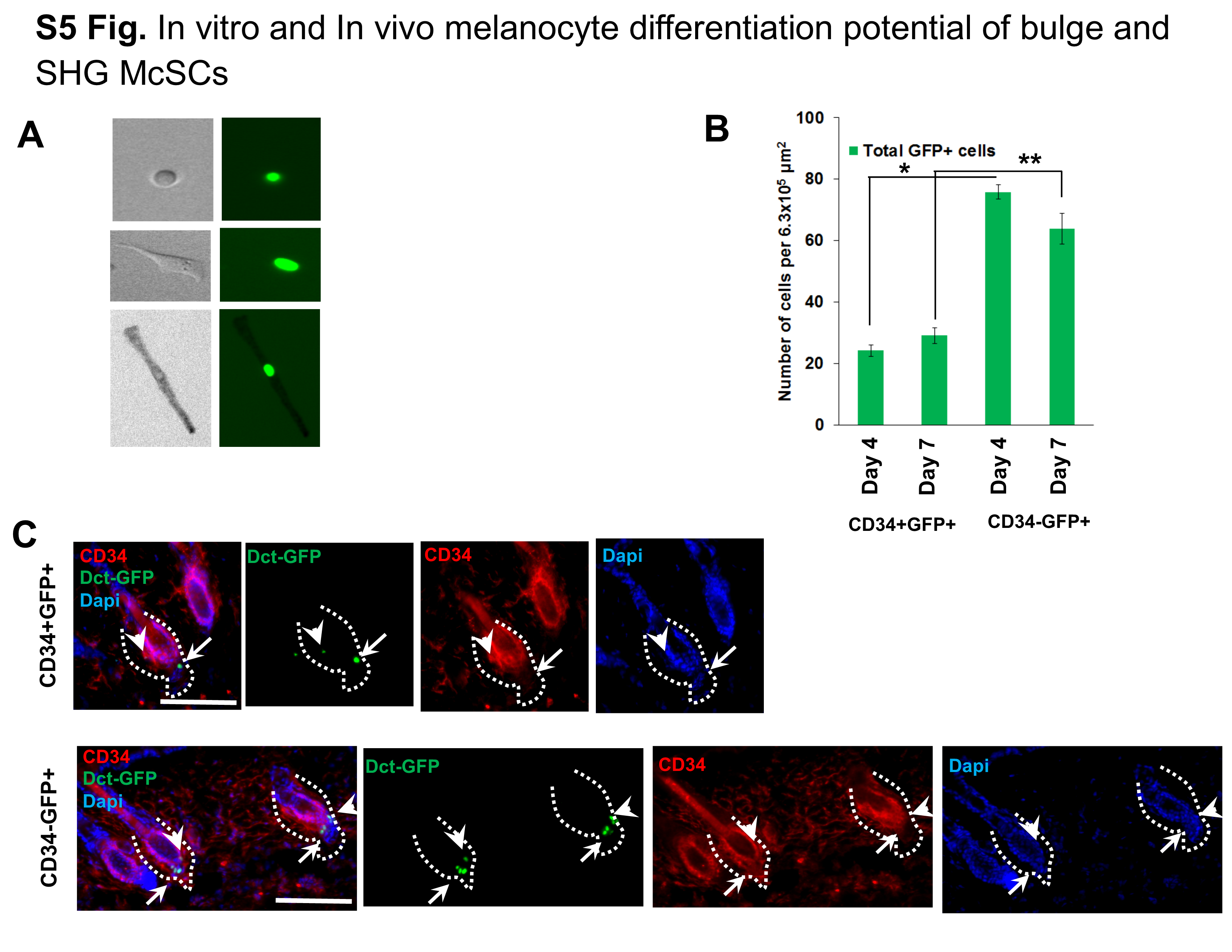

Supplement: S5 Fig — (A) & (B) Supplementary information for Fig 3B. (A) The images show three different categories used to quantify differentiation potential of bulge and SHG McSCs: Round cells (top panel), dendritic cells (middle panel) and pigmented cells (bottom panel). (B) Quantification of total GFP-expressing cells of CD34+ and CD34- McSCs when cultured in melanocyte culture condition at Day 4 and Day 7. (*P ≤ 0.01, **P Value ≤ 0.05 by ANOVA) (C) Immunofluorescence staining shows identification of GFP-expressing McSCs in CD34+ bulge (arrow head) and CD34- SHG (arrow) HFs of the skin grafts receiving either CD34+GFP+ or CD34-GFP+ McSCs at 2 months post-surgery. Scale bars: 50 μm. (TIF) [file pgen.1008034.s005.tif]

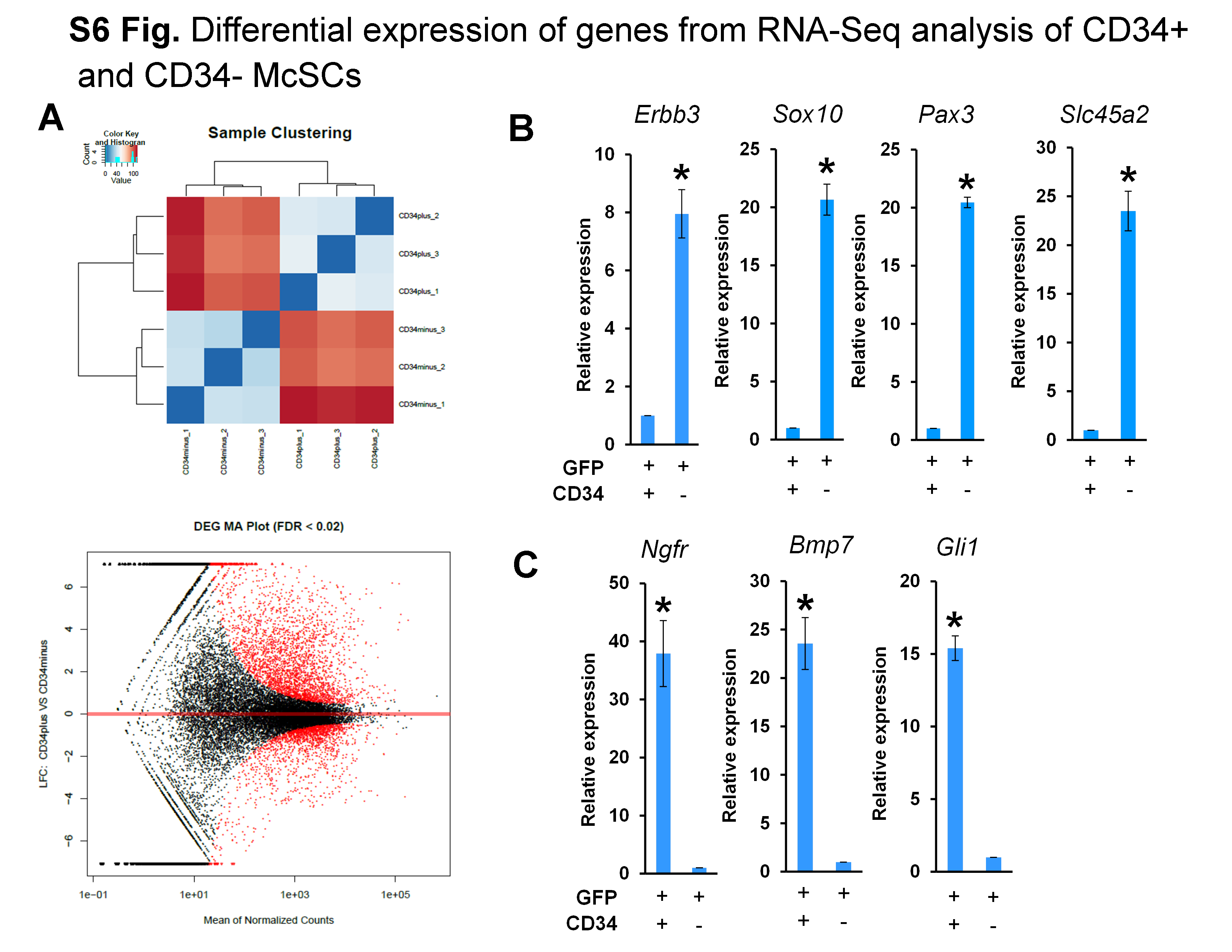

Supplement: S6 Fig — (A) Heatmap using sample clustering shows distinct differential gene expression pattern between CD34+ and CD34- McSCs. Red represents high expression of genes and blue represents low expression of genes (upper panel). MA plot of differentially expressed genes identified in CD34+ and CD34- McSCs. Data represent individual gene responses plotted as log2 fold-change CD34+/CD34- versus mean of normalized counts. FDR <0.02 was used as a cutoff to determine significant differential gene expression between two cell types. Positive and negative change represents the up-regulated genes in CD34+ and CD34- McSCs respectively and are highlighted in red (lower panel). (B) RT-PCR results show and validate higher expression of melanogenic genes and transcription factors: Pax3, Slc45a2, Erbb3 and Sox10 in CD34-/SHG McSCs (*P ≤ 0.01 by ANOVA). (C) Likewise, CD34+/bulge McSCs show higher expression of neural crest stem cell markers like Ngfr, Bmp7 and Gli1 (*P ≤ 0.01 by ANOVA). (TIF) [file pgen.1008034.s006.tif]

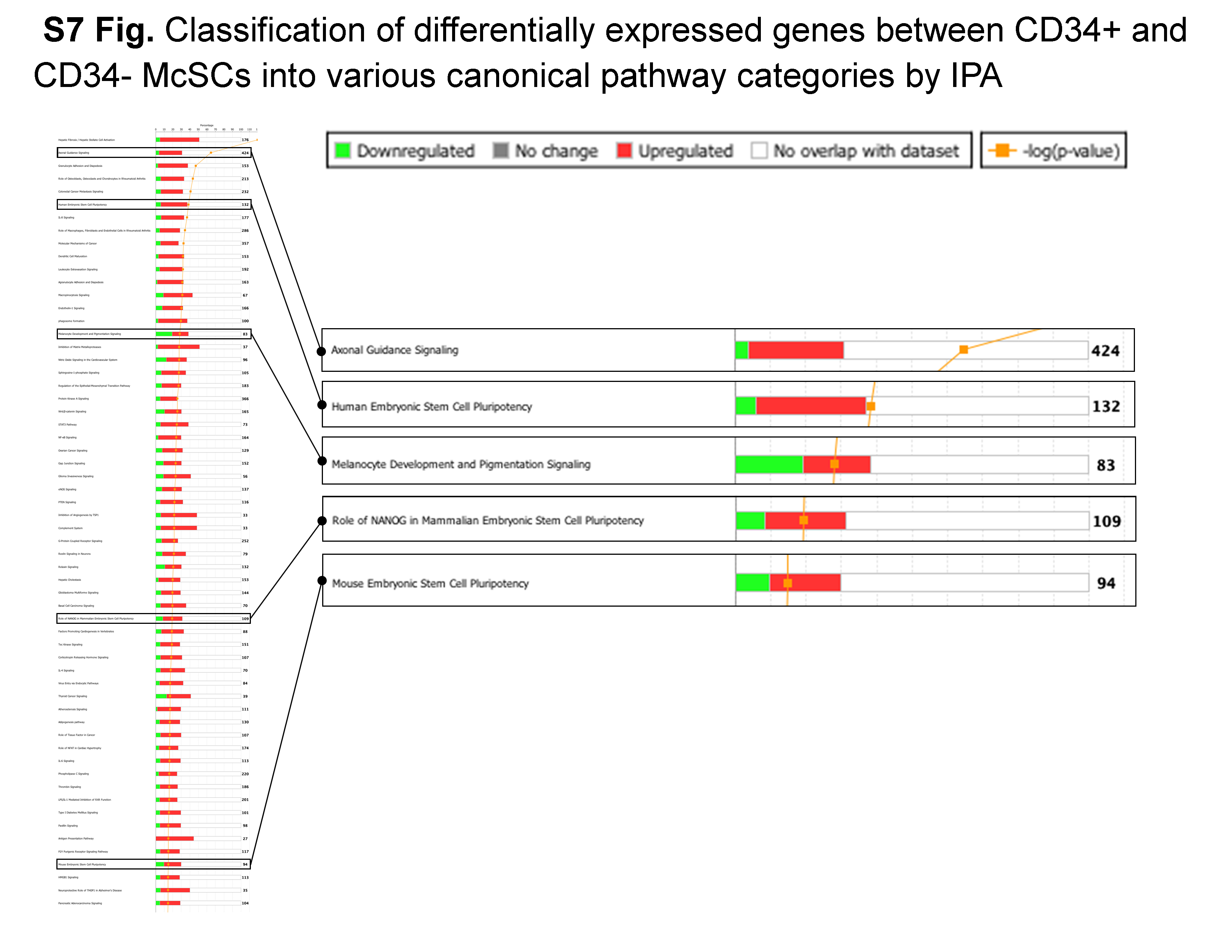

Supplement: S7 Fig — The figure depicts the highest 60 categories of the display that summarizes all 435 canonical pathways based on IPA of 3,220 differentially expressed genes (P value≤ 0.01) between CD34+ (bulge) and CD34-(SHG) McSCs. The orange line indicates the likelihood (-log(p-value)) that the genes in a specific category are differentially expressed. The stacked bar graphs show the percentage of genes that are upregulated in CD34+ McSCs (red), are downregulated in CD34+ (green) or have no overlap between the 2 McSC subsets (white). The selected top section of the graph highlights categories related to neural crest stem cells like ‘axonal guidance signaling’, ‘human embryonic stem cell pluripotency’, ‘role of NANOG in mammalian embryonic stem cell pluripotency’ and ‘mouse embryonic stem cell pluripotency’. In these categories, a higher number of genes is upregulated in CD34+ McSCs compared to CD34- McSCs. Similarly, the figure also shows the ‘melanocyte development and pigmentation signaling’ category where approximately half the genes are upregulated in CD34+ McSCs while the other half are upregulated in CD34- McSCs. (TIF) [file pgen.1008034.s007.tif]

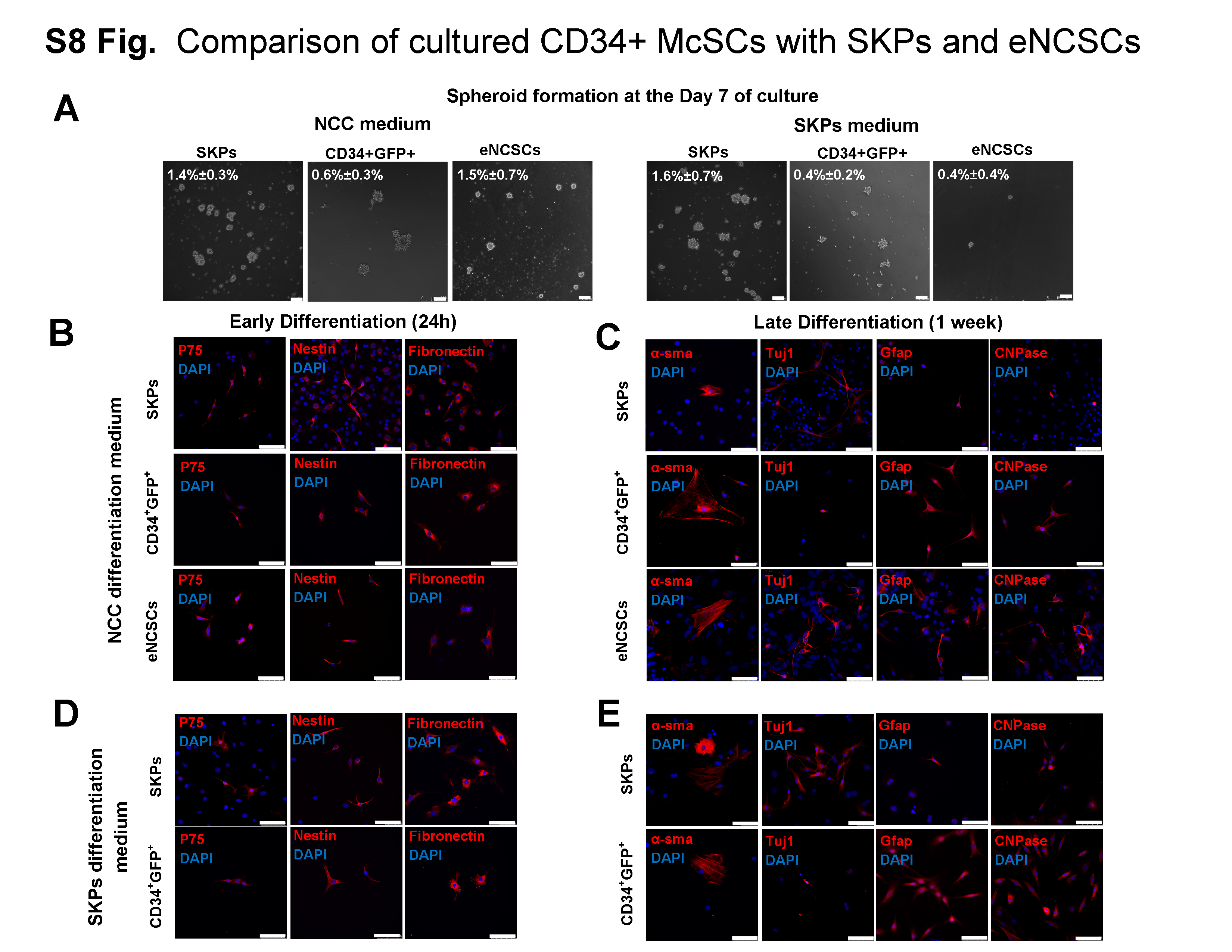

Supplement: S8 Fig — (A) CD34+ McSCs, murine SKPs and eNCSCs are grown as spheroids in NCC medium and SKP medium for 7 days. The efficiency of spheroid formation is provided at the top of each panel (N = 3). Scale bars: 100 μm. (B, C, D and E) Cells are then differentiated in neural crest differentiation medium (B and C) and SKP differentiation medium (D and E). Marker comparison is performed at early (24 hours) and late (1 week) timepoints. Immunofluorescence staining of p75, nestin and fibronectin at the early differentiation stage (B and D) and α-Sma, Tuj1, Gfap and CNPase at the late differentiation (C and E). Scale bars: 75 μm. (TIF) [file pgen.1008034.s008.tif]

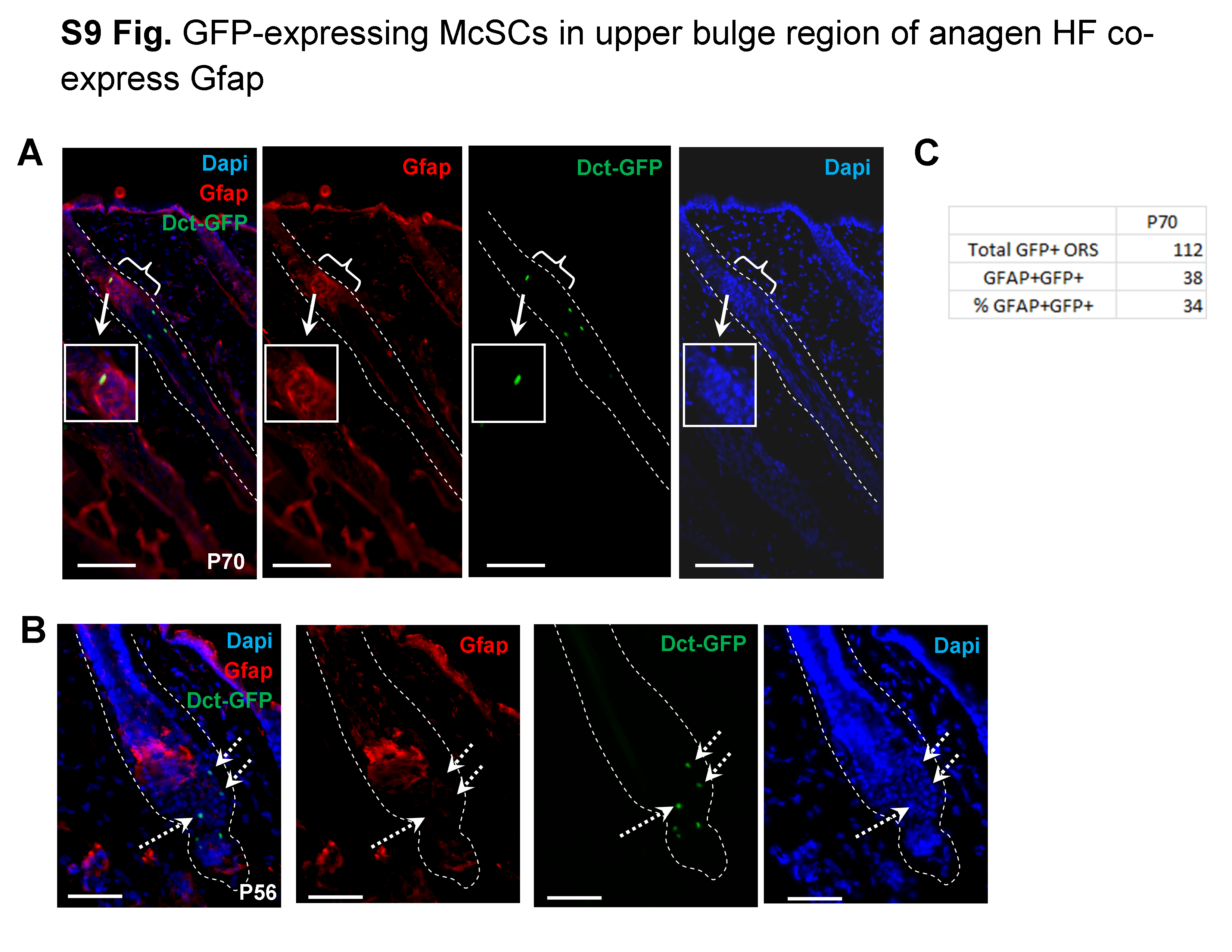

Supplement: S9 Fig — (A) GFP-expressing cells co-express Gfap (inset boxes) in the upper bulge region of growing anagen HFs in Dct-H2BGFP mice at P70. In the image, bracket depicts a distinct Gfap stained upper bulge region of elongating HF in Dct-H2BGFP mice. (B) In telogen HF of Dct-H2BGFP mice at P56, the bulge GFP-expressing cells lack Gfap expression (dotted arrow). (C) Quantification data show 34% of bulge Dct-H2BGFP-expressing cells reveal co-localization with Gfap at the onset of anagen in Dct-H2BGFP mice at P70. For this experiment, only upper and lower ORS Dct-H2BGFP-expressing McSCs were counted and mature bulb Dct-H2BGFP-expressing melanocytes were eliminated. (TIF) [file pgen.1008034.s009.tif]

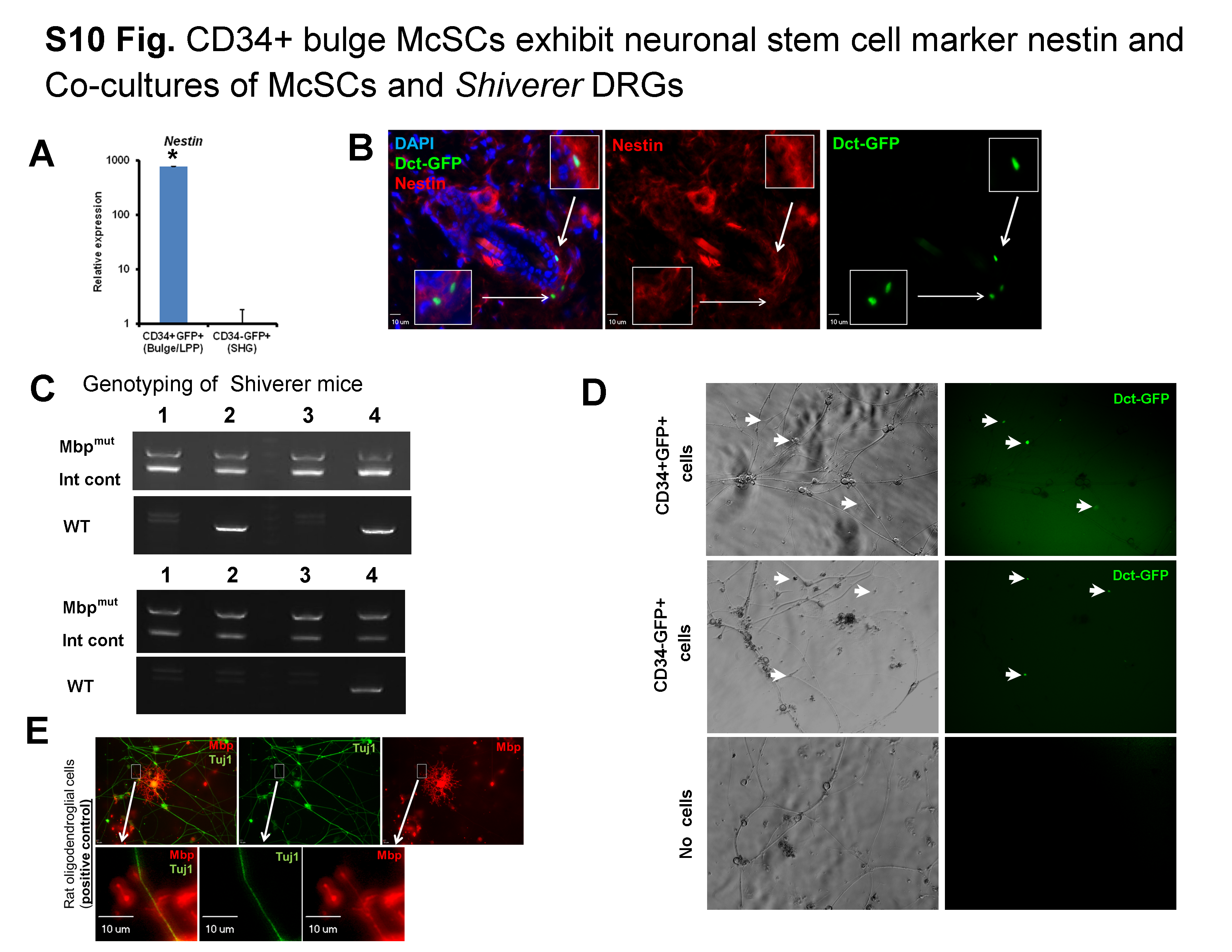

Supplement: S10 Fig — Comparison of expression of nestin mRNA (A) and protein (B) among CD34+ bulge and CD34- SHG McSCs. (C) Genotyping to identify shi/shi pups which were further used to isolate DRGs at P5 to P8. For each of two separate experiments, an individual litter was genotyped as shown in top and bottom panel. (D) A representative image for GFP-expressing cells (CD34+ or CD34- or no cells) co-cultured with neurites generated from DRGs isolated from shi/shi pups. After the localization of GFP-expressing cells in their representative cultures, cells were then fixed and analyzed with EM. (E) Co-cultures of ODCs and neonatal shi/shi DRGs as positive control. The top row depicts Mbp expressed by ODCs (left panel) and Tuj1 expressed by shi/shi axonal outgrowths (center). At bottom row, high magnification images of the region marked with white box are shown; they depict Mbp deposition along a Tuj1-expressing shi/shi axon. (TIF) [file pgen.1008034.s010.tif]

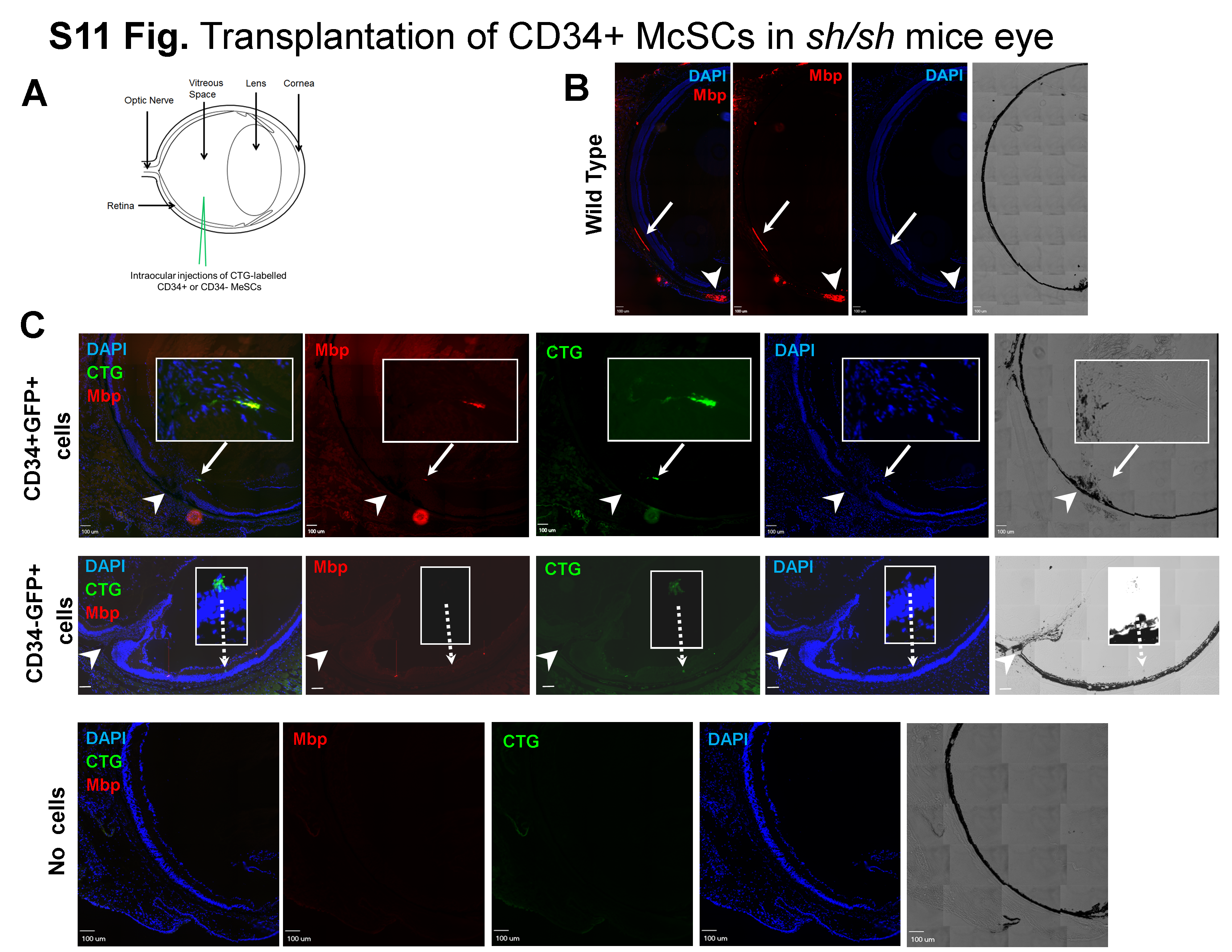

Supplement: S11 Fig — (A) Transplantation of CD34+ or CD34- McSCs, after labelling with fluorescent CTG dye, into the vitreous space of shi/shi mouse eye for 10 days. (B) Immunofluorescence for the endogenous expression of Mbp around the retinal layer (arrow) and ciliary body (arrow head) in wild type mouse eye. (C) Retinal sections of shi/shi eyes transplanted with CD34+ or CD34- McSCs show co-localization of Mbp expression in CTG-labelled CD34+ McSCs (solid arrows), whereas there is no evidence of Mbp expression in CTG-labelled CD34- McSCs (dotted arrows). Arrowhead points to the injection site in each image. The bottom panels represent a no cell-injected retinal section from control shi/shi mice. Scale bars: 100 μm. (TIF) [file pgen.1008034.s011.tif]

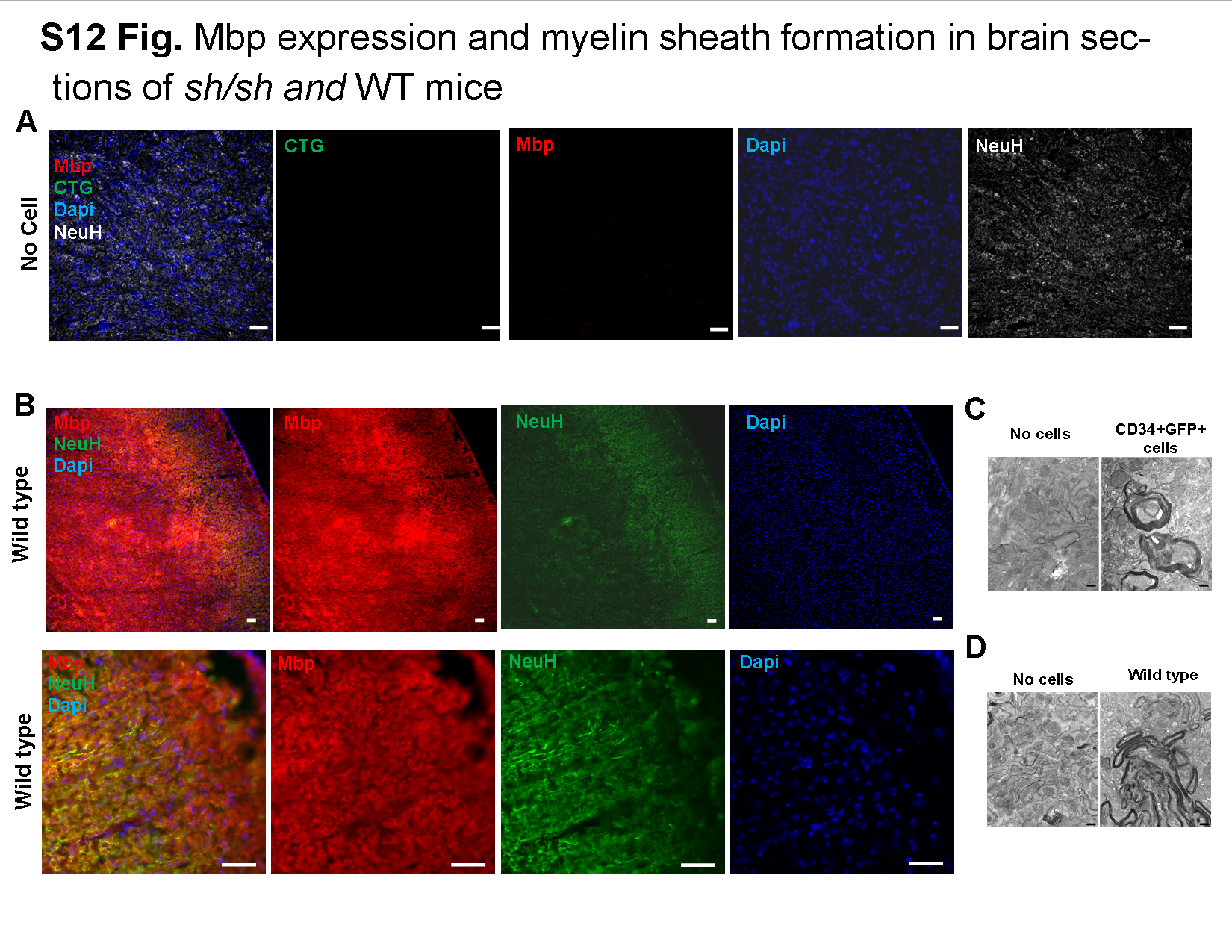

Supplement: S12 Fig — (A) Cranial sections of shi/shi brains not receiving transplanted cells show lack of Mbp expression. Scale bars: 50 μm. (B) Cranial sections of wild-type brains (positive control) show high expression of Mbp and its expression was co-localized with neurofilament H (NeuH)-expressing neurons. Upper panel is a high magnification image and bottom is a low magnification image. Scale bars: 50 μm. (C) TEM image shows dense myelin sheath image of brain sections receiving CD34+GFP+ cells whereas there is lack of myelin sheath formation in brain sections of shi/shi mice receiving no cells. (D) TEM image shows dense myelin sheath around neurons of brain sections of wild-type mice whereas lack of myelin sheath formation in brain sections of shi/shi mice receiving no cells. Scale bars: 500 nm. (TIF) [file pgen.1008034.s012.tif]
