# Supplementary material for: CD34 defines melanocyte stem cell subpopulations with distinct regenerative properties
Source: PLoS Genet. 2019 Apr 24;15(4):e1008034. doi: 10.1371/journal.pgen.1008034 (PMC6481766; doi:10.1371/journal.pgen.1008034)
Supplement: S1 Table — (DOCX) [file pgen.1008034.s013.docx]

**S1 Table: Comparison of neural crest lineage markers expressed by CD34+ McSCs and SKPs using SKP medium.**

|  | SKPs | CD34+ McSCs |
| --- | --- | --- |
| p75/Ngfr | 5.4% ± 1% | 79.1% ± 7.4% * |
| Nestin | 62.9% ± 0.2% | 71.9% ± 3.5% |
| Fibronectin | 62.3% ± 3% | 76.3% ± 4.5% |
| Tuj-1 | 9.4% ± 3% | 0.4% ± 0.03% |
| α-Sma | 10.4% ± 2.2% | 8.4% ± 2.9% |
| Gfap | 11% ± 4.3% | 29.7% ± 4.8% * |
| CNPase | 10.4% ± 4.6% | 16% ± 5.7% |

* Statistically significant differences between CD34+ McSCs and SKPs.
