# Supplementary material for: CD34 defines melanocyte stem cell subpopulations with distinct regenerative properties
Source: PLoS Genet. 2019 Apr 24;15(4):e1008034. doi: 10.1371/journal.pgen.1008034 (PMC6481766; doi:10.1371/journal.pgen.1008034)
Supplement: S1 Text — (DOCX) [file pgen.1008034.s018.docx]

**Supplemental materials and methods**

**Mice**

Generation of *Wnt1*-Cre;R26-tdTomato;Dct-H2BGFP: We first crossed *Wnt1*-Cre and R26-tdTomato mice to generate *Wnt1*-Cre;R26-tdTomato bitransgenic mice which were then crossed with *Dct*-H2BGFP bitransgenic mice to generate the *Wnt1*-Cre;R26-tdTomato;*Dct*-H2BGFP strain.

Generation of *Tyr-CreER*;R26-tdTomato;*Dct*-H2BGFP: We first crossed *Tyr-CreER* and R26-tdTomato mice to generate *Tyr-CreER*;R26-tdTomato bitransgenic mice which were then crossed with *Dct*-H2BGFP bitransgenic mice to generate the *Tyr-CreER*;R26-tdTomato;*Dct*-H2BGFP strain.

For the induction of Cre recombinase in tamoxifen-inducible *Tyr-CreER* mice, the tamoxifen was administered intraperitoneally (IP) at a dose of 75 mg/kg body weight for five consecutive days to *Tyr-CreER*;R26-tdTomato;*Dct*-H2BGFP mice. Tamoxifen was delivered to neonates at P(0-5) through IP administration to lactating mothers and to young adult mice at P(21-25) (REF;Harris ML 2013). These periods coincide with the onset of the first and second anagen.

**Immunofluorescence assay**

Dorsal skin obtained from transgenic mice at the indicated ages was embedded in OCT compound and cryosections cut with a thickness of 10µm to observe H2BGFP expression. For immunofluorescence, cryosections were fixed in 4% paraformaldehyde in PBS for 10 min at room temperature and blocked with blocking buffer (10% FBS, 1% BSA and 0.1% Triton-X) for 1h at room temperature. Primary antibodies for CD34 (rat monoclonal antibody, clone Ram34, BD Biosciences) (rabbit monoclonal antibody, Abcam), P-Cadherin (goat polyclonal antibody, R & D Systems), c-Kit (rat monoclonal antibody, Cedarlane), anti-RFP to detect tdTomato (rabbit antibody, Rockland antibodies & assay), or nestin (mouse monoclonal antibody, Chemicon) at 1:200 dilution were added and incubated at 4^o^C overnight. For immunofluorescence analysis of BrdU incorporation, 4N HCl was used to denature DNA in cryosections at room temperature for 30min and neutralized by incubation with 0.1M sodium borate for 5 minutes. Then, after blocking with MOM blocking reagent (Vector Laboratories) for 1h at room temperature, the primary antibody against BrdU (mouse monoclonal, BD Biosciences) at a 1:50 dilution was added and incubated at 1h room temperature. To detect the primary antibody, isotype-matched Cy3-conjugated secondary antibodies (Jackson Immunogenetics) or Alexa 647-conjugated secondary antibodies (Invitrogen) were added at a 1:1000 dilution and incubated for 1h at room temperature. Coverslips were mounted using mounting solution with DAPI (Vectashield, Vector Laboratories). Fluorescence was detected either using an Olympus upright fluorescence microscope, Slidebook imaging software or Leica upright fluorescence microscope, Leica application suite (LAS X) software.

For immunofluorescence detection in cultured cells, cells were washed with PBS and fixed with 4% PFA at room temperature for 10min. PBS containing 10% FBS and 1% BSA was used for blocking at room temperature for 1h. After blocking, cells were incubated with 1:200 dilution of primary antibodies for α-Sma (mouse monoclonal antibody, Sigma Aldrich), Gfap (rabbit polyclonal antibody, Dako), Tuj1 (mouse monoclonal antibody, Sigma Aldrich), K15 (mouse monoclonal antibody, Chemicon), Tyrp1 (αPEP1, rabbit polyclonal antibody, a gift from Dr. Vincent Hearing, NIH), β-Actin, or myelin basic protein (Mbp) (rat monoclonal antibody, Abcam) at 4^o^C overnight followed by incubation with Cy3-conjugated secondary antibody or Alexa 647-conjugated secondary antibody at room temperature for 1h. Coverslips were mounted using mounting solution with DAPI (Vectashield, Vector Laboratories). Fluorescence was detected using an Olympus upright fluorescence microscope, Slidebook imaging software.

For wholemount hair follicle staining, whole mount specimens of mouse tail epidermis were prepared as described previously [1, 2]. A scalpel was used to slit the tail lengthways. Skin was peeled from the tail, cut into pieces (0.5x0.5cm^2^) and incubated in 5mM EDTA in PBS at 37°C for 4h. Forceps were used to gently peel the intact sheet of epidermis away from the dermis and the epidermal tissue was fixed in 4% formal saline (Sigma) for 2h at room temperature. Fixed epidermal sheets were stored in PBS containing 0.2% sodium azide at 4°C for up to 8 weeks prior to labelling. For immunofluorescence staining of wholemount hair follicle, epidermal sheets were blocked and permeabilized by incubation in blocking buffer containing 10% FBS, 1% BSA and 0.5% Triton-X in PBS for 30 minutes. Primary antibodies were diluted in the blocking buffer and tissue was incubated overnight at 4^o^C with gentle agitation. Epidermal wholemounts were then washed for at least 4h in PBS, changing the buffer several times. Incubation with secondary antibodies was performed in the same way. Samples were rinsed in distilled water and mounted in mounting solution with DAPI.

**RNA extraction, cDNA synthesis and qRT-PCR**

Total RNA was extracted from sorted cells using RNeasy Micro Kit (Qiagen) as per manufacturer’s protocol. Following transfer to a spin column, incubation with a DNase I, and washing, the spin column membrane was eluted with 14µl RNase free water. The quantity and quality of isolated RNA was determined with an Agilent 2100 Bioanalyzer using RNA PicoChips (Agilent). To synthesize first-strand cDNA from total RNA we used the SuperScript III First-Strand Synthesis System for RT-PCR (Invitrogen). Total RNA was incubated with random hexamer primer along with dNTP at 65^o^C for 5min and added to a mixture of reverse transcriptase containing reaction buffer incubated first at 25^o^C for 10min, then at 50^o^C for 50min, and the reaction terminated by heating at 85^o^C for 5 min. qRT-PCR analysis for the differential gene expression among the sorted cell population was determined using LightCycler 480 SYBER Green I Master (Roche) and running them on LightCycler 480 instrument (Roche).

**RNA-seq and differential gene expression analysis**

RNA was isolated using Qiagen RNeasy Micro Kit, which include DNase treatment. 30 ng of RNA per sample was used as an input for the Nugen Ovation RNA-Seq V2 System. This system generates linearly amplified cDNA, and synthesis of cDNA from ribosomal RNA is suppressed. Amplifications typically result in microgram amounts of SPIA cDNA, which was then fragmented to an average length of 500 bp with a Covaris S2 System using the manufacturer’s recommended settings. 100 ng of fragmented cDNA was used as input for the Ovation Ultralow Library System. Samples are subjected to end repair, ligation, and PCR amplification to add Illumina adapter sequence and indexes. The system typically yields >1 µg of enriched library, which then was sequenced on 2X100 base pair Illumina HiSeq 2000 run.

An Ergatis-based [3] RNA-Seq analysis pipeline was used for analyzing the paired-end Illumina sequencing reads. FASTQC software (v.0.11.5) (http://www.bioinformatics.babraham.ac.uk/projects/fastqc) was used to evaluate the quality of the raw sequencing reads for each sample. Based on the quality checks, the reads were trimmed to remove adapters and low quality reads using the FASTX-toolkit (v.0.0.13) (http://hannonlab.cshl.edu/fastx_toolkit/index.html). The Mus musculus genome (version GRCm38, release 83) downloaded from Ensembl (http://useast.ensembl.org/Mus_musculus/Info/Index) was used for the RNA-seq analysis. The sequencing reads were mapped to the Mus musculus genome using TopHat (v1.4.0), which uses Bowtie (v0.12.9) for read mapping [4-6]. The BAM files containing aligned reads were processed and evaluated using SAMtools (v1.4.1).

The number of reads mapping to each gene in the reference genome annotation was counted using HTSeq (v0.4.7) [7]. The DESeq R/Bioconductor package (v1.10.1) was used to normalize the raw counts for each sample to account for differences in library sizes, estimate gene-level dispersion, and then test for differential gene expression with the negative binomial model [8]. DESeq uses the false discovery rate (FDR) of Benjamini and Hochberg’s approach to adjust p-values for multiple testing [9]. The filter cutoffs for determining if differences in gene expression were significant were a false discovery rate (FDR) < 0.02 and an absolute log2 fold change of ≥ 1.

Variance stabilizing transformed (VST) counts from DESeq were used as input for Euclidean distances-based hierarchical clustering to evaluate sample-sample distances and to explore gene expression patterns between CD34+ samples and CD34- samples and to visualize them using heatmaps. The heatmaps that are displayed in the figures and supplementary figures were generated by the heatmap.2() function. To understand the biological context of the differences between CD34+ and CD34-, pathway analysis was conducting using Ingenuity Pathway Analysis platform (IPA, QIAGEN Redwood City, www.qiagen.com/ingenuity) with the list of differentially expressed genes as input.

**Electron microscopy**

The preparation of DRG co-cultures for electron microscopy was achieved by removing the medium and washing twice with the sodium cacodylate buffer (0.1M sodium cacodylate + 3mM CaCl2; PH 7.4). The DRG cells from co-cultures were fixed with 3% glutaraldehyde in sodium cacodylate buffer for 30min at room temperature and then stored at 4^o^C. After buffer rinse, samples were postfixed in 1% osmium tetroxide in buffer for 1h on ice in the dark.  Following a distilled H_2_O rinse, plates were stained with 2% aqueous uranyl acetate (0.22µm filtered, 1h in the dark), dehydrated in a graded series of ethanol and embedded in Eponate 12 (Ted Pella) resin.  Samples were polymerized at 37^o^C for 2-3 days before moving to 60^o^C overnight. Thin sections, 60 to 90nm, were cut with a diamond knife on the Reichert-Jung Ultracut E ultramicrotome and picked up with 2x1mm copper slot grids.  Grids were stained with 2% uranyl acetate in 50% methanol and lead citrate at 4^o^C and observed with a Philips CM120 at 80kV.  Images were captured with an AMT CCD XR80 (4K x 4K) camera.

***In vivo* assay for hair pigmentary regeneration**

Skin specimens obtained from Mitf^Mi-wh/Mi-wh^ mutant mouse neonates at P0 to P2, were used as donor skin and nude mice as recipients for the in vivo hair pigmentary regeneration assay. Donor skin specimens were incubated in 2% EDTA at 37^O^C for 2 hours and, using a 1 cc tuberculin syringe with a 30 gauge needle, 30,000 FACS-sorted cells injected between the dermis and epidermis of EDTA-treated skin, raising a visible blister. Injected skin specimens were kept at room temperature for 1 hour, then moved to 4^O^C overnight. The next morning, injected skin was grafted onto *nu/nu* mouse recipients. Hair growth and de novo follicular pigmentation could be observed about 2 weeks later.

**Statistical analysis**

The statistical analysis for the qRT-PCR of differential gene expression among sorted cells (Figure 2C, D; S6B, C; and S10A) was performed using one-way ANOVA (Prism). For the quantification data of the bulge and SHG melanocyte precursor cell potential to produce pigmented melanocytes in melanocyte differentiation medium at 4^th^ and 7^th^ day (Figure 3B), two-way ANOVA was applied, n=5 independent determinations for bulge and SHG melanocyte precursor cell images. The statistical analysis for the quantification of bulge/LPP McSCs and SHG melanocyte precursor cells ability to form larger spheroids when cultured in neural crest stem cell medium measured at 2^nd^, 4^th^, 6^th^ and 8^th^ day (Figure 4D) was determined using two-way ANOVA, n=5 independent determinations for bulge and SHG melanocyte precursor cell images. The statistical analysis for quantification of pigmented region of engrafted skin receiving CD34- McSCs, or CD34+ McSCs, or no cells (Figure 3E) was determined using one-way ANOVA, n=5 mice. The statistical analysis for quantification of co-localization of Mbp expression in CTG-labelled CD34+ McSCs in cranial sections of shi/shi brains transplanted with CD34+ or CD34- McSCs (Figure 6E) was determined using one-way ANOVA, n=5 mice.

**List of Primers**

The primer sequences for mouse *Dct*, *Tyr*, *Tyrp1*, *Pmel17* [10], *Gapdh* [11], *Slc45a2* [12], *Pax3* [13], *Sox10* [14], *Erbb3*, *Gli* [15], *Ngfr* [16], and *Bmp7* [17] were designed as described previously.

*Dct*

Forward: 5’ – TTCGCAAAGGCTATGCGC – 3’

Reverse: 5’ GTTACTACCCAGGTCAGGCCAG – 3’

*Tyr*

Forward : 5’ – CGGCCAACGATCCCATT – 3’

Reverse: 5’ – TGCCTTCGCAGCCATTG – 3’

*Tyrp1*

Forward: 5’ – GTGTTCCCTAGCTCAGTTCTCTGG – 3’

Reverse: 5’ – TCCTCTGACTGATACCTT – 3’

*Pmel17*

Forward: 5’ – TCCAGGAATCAGGACTGGCTTGGT– 3’

Reverse: 5’ – GTGAAGGTTGAACTGGCGTG - 3’

*Gapdh*

Forward: 5’ – TGCAGTGGCAAAGTGGAGATTGTTG – 3’

Reverse: 5’ – TGTAGCCCAAGATGCCCTTCAG – 3’

*Cytokeratin 14*

Forward: 5’ – ATCGAGGACCTGAAGAGCAA – 3’

Reverse: 5’ – GGCTCTCAATCTGCATCTCC – 3’

*P-Cad*

Forward: 5’ – ACAGCATCACAGGGCCTGGC – 3’

Reverse: 5’ – TGGCTCCTTCGGCTCTTGGC – 3’

*Slc45a2*

Forward: 5′ - GCCGACTGACACCCATACC - 3'

Reverse: 5′- CTGTGCATGACAAGTCTCCC - 3'

*Pax3*

Forward: 5' - ACTACCCAGACATTTACACCAGG - 3'

Reverse: 5'- AATGAGATGGTTGAAAGCCATCAG - 3'

*Erbb3*

Forward: 5′ - TACCAACTCCAGCCATGCTC - 3'

Reverse: 5′- CACGATGTCCCTCCAGTCAA - 3'

*Sox10*

Forward: 5′-CAGTCCGGCAAGGCAGACCC-3′

Reverse: 5′-GCAGGTATTGGTCCAGCTCAGTCAC-3′

*Gli1*

Forward: 5′ - TGAGCATTATGGACAAGTGCAGGT - 3'

Reverse: 5′- ATTGAGGCAGGGTGCCAATC - 3'

*Ngfr*

Forward: 5’ – GACTAACCTAGGCCACCCAA – 3’

Reverse 5’ – CAGACGTCGTTTCCAGATGT – 3’

*Bmp7*

Forward: 5' - TACGTCAGCTTCCGAGACCT - 3'

Reverse: 5'- GGTGGCGTTCATGTAGGAGT - 3'

***shi/shi* genotyping primers**

The *shi/shi* genotyping was performed as described by Jackson laboratory.

Mutant primer (303bp)

Mbp^shi^ Fwd

ACC GTC CTG AGA CCA TTG TC

Mbp^shi^ Rev

GTG CTT ATC TAG TGT ATG CCT GTG

Internal positive control (200bp)

Control Fwd

CAA ATG TTG CTT GTC TGG TG

Control Rev

GTC AGT CGA GTG CAC AGT TT

WT *shi/shi* primer (411bp) {overlaps 3^rd^ exon and 3^rd^ intron}

WT^shi^ Fwd

GGCCGGACCCAAGATGAAAAC

WT^shi^ Rev

TGTTGGCCTAAAGCACCCTAC

**References**

1.     Braun KM, Niemann C, Jensen UB, Sundberg JP, Silva-Vargas V, et al. (2003) Manipulation of stem cell proliferation and lineage commitment: Visualisation of label-retaining cells in wholemounts of mouse epidermis. Development 130(21): 5241-5255. 10.1242/dev.00703 [doi].

2.     Estrach S, Ambler CA, Lo Celso C, Hozumi K, Watt FM. (2006) Jagged 1 is a beta-catenin target gene required for ectopic hair follicle formation in adult epidermis. Development 133(22): 4427-4438. dev.02644 [pii].

3.     Orvis J, Crabtree J, Galens K, Gussman A, Inman JM, et al. (2010) Ergatis: A web interface and scalable software system for bioinformatics workflows. Bioinformatics 26(12): 1488-1492. 10.1093/bioinformatics/btq167 [doi].

4.     Langmead B, Trapnell C, Pop M, Salzberg SL. (2009) Ultrafast and memory-efficient alignment of short DNA sequences to the human genome. Genome Biol 10(3): R25-2009-10-3-r25. Epub 2009 Mar 4. 10.1186/gb-2009-10-3-r25 [doi].

5.     Trapnell C, Pachter L, Salzberg SL. (2009) TopHat: Discovering splice junctions with RNA-seq. Bioinformatics 25(9): 1105-1111. 10.1093/bioinformatics/btp120 [doi].

6.     Kim D, Pertea G, Trapnell C, Pimentel H, Kelley R, et al. (2013) TopHat2: Accurate alignment of transcriptomes in the presence of insertions, deletions and gene fusions. Genome Biol 14(4): R36-2013-14-4-r36. 10.1186/gb-2013-14-4-r36 [doi].

7.     Anders S, Pyl PT, Huber W. (2015) HTSeq--a python framework to work with high-throughput sequencing data. Bioinformatics 31(2): 166-169. 10.1093/bioinformatics/btu638 [doi].

8.     Anders S, Huber W. (2010) Differential expression analysis for sequence count data. Genome Biol 11(10): R106-2010-11-10-r106. Epub 2010 Oct 27. 10.1186/gb-2010-11-10-r106 [doi].

9.     Benjamini, Y. and Hochberg, Y. (1995) **Controlling the false discovery rate: A practical and powerful approach to multiple testing**. 57, No. 1: 289.

10.     Lanning JL, Wallace JS, Zhang D, Diwakar G, Jiao Z, et al. (2005) Altered melanocyte differentiation and retinal pigmented epithelium transdifferentiation induced by Mash1 expression in pigment cell precursors. J Invest Dermatol 125(4): 805-817. JID23819 [pii].

11.     Villareal MO, Han J, Yamada P, Shigemori H, Isoda H. (2010) Hirseins inhibit melanogenesis by regulating the gene expressions of mitf and melanogenesis enzymes. Exp Dermatol 19(5): 450-457. 10.1111/j.1600-0625.2009.00964.x [doi].

12.     Bartolke R, Heinisch JJ, Wieczorek H, Vitavska O. (2014) Proton-associated sucrose transport of mammalian solute carrier family 45: An analysis in saccharomyces cerevisiae. Biochem J 464(2): 193-201. 10.1042/BJ20140572 [doi].

13.     Costamagna D, Mommaerts H, Sampaolesi M, Tylzanowski P. (2016) Noggin inactivation affects the number and differentiation potential of muscle progenitor cells in vivo. Sci Rep 6: 31949. 10.1038/srep31949 [doi].

14.     Motohashi T, Kitagawa D, Watanabe N, Wakaoka T, Kunisada T. (2014) Neural crest-derived cells sustain their multipotency even after entry into their target tissues. Dev Dyn 243(3): 368-380. 10.1002/dvdy.24072 [doi].

15.     Mizukoshi K, Koyama N, Hayashi T, Zheng L, Matsuura S, et al. (2016) Shh/ptch and EGF/ErbB cooperatively regulate branching morphogenesis of fetal mouse submandibular glands. Dev Biol 412(2): 278-287. 10.1016/j.ydbio.2016.02.018 [doi].

16.     Tang Z, Arjunan P, Lee C, Li Y, Kumar A, et al. (2010) Survival effect of PDGF-CC rescues neurons from apoptosis in both brain and retina by regulating GSK3beta phosphorylation. J Exp Med 207(4): 867-880. 10.1084/jem.20091704 [doi].

17.     Tsuji K, Bandyopadhyay A, Harfe BD, Cox K, Kakar S, et al. (2006) BMP2 activity, although dispensable for bone formation, is required for the initiation of fracture healing. Nat Genet 38(12): 1424-1429. ng1916 [pii].
